# Intervention Strategies for Healthcare Workers to Promote Vaccine Uptake in Ethnic Minority Populations: A Systematic Review of Behaviour Change Techniques

**DOI:** 10.3390/healthcare14060749

**Published:** 2026-03-16

**Authors:** Winifred Ekezie, Aaisha Connor, Emma Gibson, Angel M. Chater, Kamlesh Khunti, Atiya Kamal

**Affiliations:** 1 Diabetes Research Centre, University of Leicester, Leicester LE5 4PW, UK; 2 Centre for Ethnic Health Research, University of Leicester, Leicester LE5 4PW, UK; 3 Center for Health and Society, Aston University, Birmingham B4 7ET, UK; 4 Department of Psychology and Counselling, Birmingham City University, Birmingham B4 7BD, UK; 5 Centre for Health, Wellbeing and Behaviour Change, University of Bedfordshire, Bedford MK41 9EA, UK; 6 Centre for Behaviour Change, University College London, 1-19 Torrington Place, London WC1E 7HB, UK

**Keywords:** vaccine uptake, ethnic minorities, barriers, facilitators, interventions, service delivery, behaviour change techniques, behaviour change wheel, healthcare workers

## Abstract

**Background/Objectives:** Healthcare workers (HCWs) have a crucial role in addressing vaccine hesitancy in ethnic minority populations as they are a trusted source of information. The aim of this systematic review is to synthesise and evaluate behaviour change techniques (BCTs) and strategies in interventions aimed at HCWs to promote vaccine uptake among ethnic minority populations. **Methods:** The literature was systematically searched in peer-reviewed databases and the grey literature. Studies were included if they reported interventions for respiratory and routinely recommended vaccine-preventable diseases which were delivered by HCWs to increase vaccine uptake in ethnic minority groups. Interventions were coded using the Behaviour Change Wheel (BCW) and BCT Taxonomy. **Results:** From 7250 records identified, 14 studies were included in the review. Vaccines targeted by interventions included influenza, pneumococcal disease, pertussis, tetanus, diphtheria, meningitis and hepatitis B. Seven BCW intervention types, six policy options and 22 BCTs were identified. Main intervention types used were persuasion, enablement and education. Effective interventions had multi-components and were tailored to specific populations. Staff training to improve vaccine recommendation and dialogue with patients, and prompts/cues were associated with positive effects, but there was no strong evidence to recommend one specific intervention strategy over another as effectiveness was linked to a multitude of BCTs and intervention types. **Conclusions:** Several strategies aimed at HCWs can be used and tailored to increase vaccine uptake among ethnic minority communities; however, this does not address all issues related to low vaccine uptake. While HCWs are necessary, without system-level enablement, they cannot fully address barriers to vaccine uptake.

## 1. Introduction

Vaccine hesitancy, which is the delay in acceptance or refusal of vaccination despite the availability of vaccination services, is considered a major threat to global health [1]. Wider determinants of vaccine uptake and hesitancy include gender [2,3], working conditions, education levels, and trust in healthcare services [3,4,5,6,7,8].

Low vaccine uptake has been reported in some ethnic minority populations in western contexts due to low vaccine confidence, lack of information about vaccine safety, misinformation, inaccessible communications and logistical issues [9,10,11,12,13,14,15]. Ethnicity intersects with other risk factors such as age, deprivation and comorbidities, and if barriers are not addressed by vaccination programmes, it has the potential to exacerbate existing health inequalities [10]. There are several interventions that take these factors into consideration, with the aim of improving vaccine uptake among ethnic minority groups, but little is known about the role and impact of healthcare workers (HCWs) in these interventions.

HCWs hold a unique role within vaccination programmes as they are considered one of the most trusted sources for vaccine communications more broadly and in many ethnic minority communities [4,16,17]. Barriers to vaccine acceptance and uptake among ethnic minority groups can be reduced by increasing HCWs knowledge and understanding of ethnic minority communities [18,19]. This is vital, as HCWs act as the bridge between health systems, research evidence, and patient outcomes; thus, HCWs are an essential component for the successful delivery and sustained uptake of health services [20].

Vaccine intervention strategies are required to address systemic, societal and individual barriers and to promote vaccine uptake [21]. Intervention development guidelines recommend the use of evidence-based behaviour change strategies [22,23], which require understanding the behavioural elements of particular interventions that would improve vaccine uptake.

The Behaviour Change Wheel [24] and Behaviour Change Technique Taxonomy (BCTTv1) [25] are frameworks for describing and designing intervention content and identifying the influence of intervention components on behaviours (mechanism of action) and how they occur (change process) [26]. The Behaviour Change Wheel (BCW) [24] has a central system at the ‘hub’ of the wheel that comprises three components that influence behaviour: capability, opportunity, and motivation, known as the COM-B model. Within the COM-B model, there are six dimensions: physical and psychological capability (e.g., knowledge, skills, memory and ability to regulate behaviour); social and physical opportunity (external factors that make execution of a behaviour possible such as social influences and environmental context); and automatic and reflective motivation (internal processes that energise behaviour such as beliefs, intentions, identity, reinforcement and emotions). The second layer of the BCW includes nine intervention types (education, persuasion, incentivisation, coercion, training, enablement, modelling, environmental restructuring and restrictions), and the outer layer has seven policy options that facilitate behaviour change (communication/marketing, guidelines, fiscal measures, regulation, legislation, environmental/social planning and service provision) [23,24]. The BCTTv1 consists of 93 BCTs which are clustered into 16 groups that can enable behaviour change (see Supplementary File S1). BCTs are ‘active ingredients’ of interventions designed to support an individual to change or regulate their behaviour by influencing mechanisms of action and/or minimising the barriers to behaviour change [27]. Classifying BCTs used in interventions can help to understand how and why interventions achieve their effects specific to particular behaviours, populations and contexts. This information enables the tailoring of interventions that are more likely to include effective components and better explain intervention effects [24,25,26,27].

It is important to identify interventions and strategies that can support HCWs on how to optimise vaccine uptake and reduce hesitancy among ethnic minority communities. Understanding these factors will inform the development of future interventions that address inequalities and provide equitable measures to support vaccination programmes that meet the health needs of a diverse range of communities.

### Review Questions

This systematic review sought to answer the following questions:

1. What intervention strategies targeting HCWs can increase vaccine uptake in ethnic minority groups?
2. What BCTs are included within vaccination programmes delivered by HCWs that are designed to increase vaccine uptake in ethnic minority groups?

## 2. Methods

This systematic review followed the Preferred Reporting Items for Systematic Reviews and Meta-Analyses (PRISMA) guidelines [28]. The protocol was pre-registered on the International Prospective Register of Systematic Reviews (PROSPERO ID: CRD42021239010) [29].

### 2.1. Search Strategy

The search strategy was applied to the following databases: MEDLINE, EMBASE, CINAHL, EBSCOhost, and PsycInfo. Hand searching was conducted over the last nine years in two key journals (Vaccine and Vaccines). These databases and journals were selected based on their coverage of public health and vaccination topics. For the grey literature, a search was conducted through the first 10 pages of Google Scholar and pre-print databases (SocArXiv, MedRXiv, PsyRXiv, and SSRN). The search terms were based on a combination of keywords for three key concepts: “vaccine hesitancy” AND “ethnic minority groups” AND “intervention”. Within each concept, keywords were combined with Boolean search operators (see Supplementary File S2). Table 1 shows the keywords included in the search strategy for each concept.

Searches of databases had no date restriction and included papers from inception to 24 March 2021; the search was updated and included papers up to 29 August 2025. Studies not captured by the database search engines were identified through bibliometric cross-referencing.

### 2.2. Eligibility Criteria

Included studies could report interventions for respiratory and routinely recommended vaccine-preventable diseases; for example, influenza, pneumonia, and diphtheria-pertussis-tetanus (DPT), which were delivered by HCWs to increase vaccine uptake in ethnic minority groups. Vaccine-preventable, vector-borne, sexually transmitted infectious diseases and non-routinely recommended vaccines were excluded in the search terms (i.e., NOT “HPV or malaria or typhoid or cholera”) (see Supplementary File S2—OVID Medline search terms) as these vaccines include different considerations to many routinely recommended vaccines. Studies on COVID-19 vaccines were excluded as the original search was developed before widespread availability of the COVID-19 vaccine. If a study included groups of diseases that were in the included and excluded categories, e.g., influenza and COVID-19, only information related to respiratory non-COVID-19 and routinely recommended vaccines were extracted.

Using PICOS (Population, Intervention, Comparator, Outcome, Study Design), the following inclusion and exclusion criteria were used:

- Population: studies that included people from ethnic minority groups, with evidence of HCW role and activities; studies were excluded if they had a majority White ethnicity population (i.e., studies with ≥50% White ethnic sample size) and/or did not explicitly indicate the activities of HCWs. Studies with ≥50% White participants were excluded to ensure majority representation of ethnic minorities and to increase the likelihood that the findings were generalised to the ethnic minority population.
- Interventions: reported interventions which included specific strategies provided to HCWs designed to improve vaccination services and uptake, focusing on respiratory and routinely recommended vaccine-preventable diseases; studies were excluded that did not provide details of the interventions.
- Comparator: included any reported comparator such as pre-intervention data, alternative intervention, or control group.
- Outcomes: studies were included if they reported vaccine behaviour-related data (intention, behaviour and uptake) of patients from ethnic minority backgrounds after implementation of the intervention.
- Study Design: all study designs, including quantitative and qualitative, were included except case studies and case series.

Only peer-reviewed articles in the English language were included. Papers were excluded if there was no empirical data, if they reported conference proceedings, or were not in English.

## 3. Screening

Each reference was uploaded to the Rayyan review manager, an app with semi-automation that helped with the screening process [30]. After the automatic removal of all duplicates, two investigators (WE and AC) conducted screening in different phases. One reviewer (WE) screened 100% of the studies, and the second reviewer (AC) blindly screened 50% of the studies at each phase to ensure consistency. The abstracts and titles were first screened to identify studies that reported interventions with ethnic minority populations for inclusion. Next, studies that met the initial inclusion criteria underwent full-text screening to identify those that reported HCW involvement and strategies in interventions involving ethnic minority populations. Following this, the outcome criteria of interest were also screened in studies reporting HCW strategies in interventions to support ethnic minority populations. The reference lists of all papers included in the synthesis were reviewed for additional articles. Discrepancies across the different screening phases were resolved through discussion among WE, AC, and AK.

### 3.1. Data Extraction

#### 3.1.1. Intervention Study Details

Data were extracted by WE and AC separately. Both reviewers independently extracted 50% of all studies and 10% of the sample from each individual’s extraction, and 10% of each reviewer’s sample was quality-checked by the other reviewer (WE or AC). Approximately 90% consistency was observed. All discrepancies were resolved through discussions between WE, AC and AK. For each study, the following data were extracted if available: vaccine focus; study information (including country of study and design); participant characteristics (sample size, ethnicity, age, etc.); intervention details (intervention components, outcomes, etc.); and vaccine coverage or uptake, hesitancy, barriers and facilitators.

#### 3.1.2. BCW and BCT Intervention Details

Two reviewers (WE and EG) read the intervention descriptions to identify the BCW components, intervention types and policy options reflected within each intervention [23,24]. The BCTTv1 was used to identify BCTs from the 93 BCTs used in each intervention which were then categorised into 16 groups. Intervention content was also mapped onto the BCW, intervention types and policy options [25]. This information (BCW and BCT details) was entered onto a standardised data extraction form. All included studies were coded for the BCTs and BCW by one author (WE), these were all reviewed by a second author (EG), and discrepancies were resolved by a third author (AK) to reach consensus.

### 3.2. Risk of Bias

Risk of bias was measured using the AXIS critical appraisal tool [31] for cross-sectional studies and Critical Appraisals Skills Programme (CASP) guidelines for other study designs [32]. The AXIS critical appraisal tool included 20 questions to address study design, reporting quality, and the risk of bias in cross-sectional studies. CASP guidelines for cohort studies, randomised controlled trials, and qualitative research included questions to assess appropriateness of study design, methodology, and results. Each reviewer (WE and AC) assessed 50% of included studies, 10% of these were reviewed by a second reviewer, with discrepancies being resolved by a third author (AK). Studies were rated low, moderate or high. For each study design, the proportion of positive assessments was used to determine the quality of each study. For example, fewer than 11 positive scores using the AXIS critical appraisal tool was considered low quality, 11–16 = moderate, and a score of 17 or higher was high quality (Supplementary File S3).

### 3.3. Synthesis Method

Findings from the included studies were entered into tables and descriptively synthesised. The analysis explored the variation in vaccines reported, study design information, and intervention details and outcomes. Effect sizes of the outcomes were not accessed due to wide variation in the details reported; this included differences in the measure of effects being used, lack of analysis, heterogeneity of the population samples and insufficient data reporting the same outcome across the studies. Due to the vast differences in the types of interventions, components, measurements and reported outcomes, a meta-analysis could not be conducted.

The specific strategies used in each intervention were identified, extracted and mapped to the related BCW and BCT components. In addition, factors that influenced the intervention implementation and outcomes, such as information on uptake, hesitancy, barriers and facilitators to uptake, and challenges experienced while implementing the intervention, were extracted and analysed. Finally, the recommendations suggested by the study authors on approaches to improve similar interventions and other areas that need further exploration were summarised and grouped according to the related BCTs.

## 4. Results

### 4.1. Search Results

**Figure 1:**
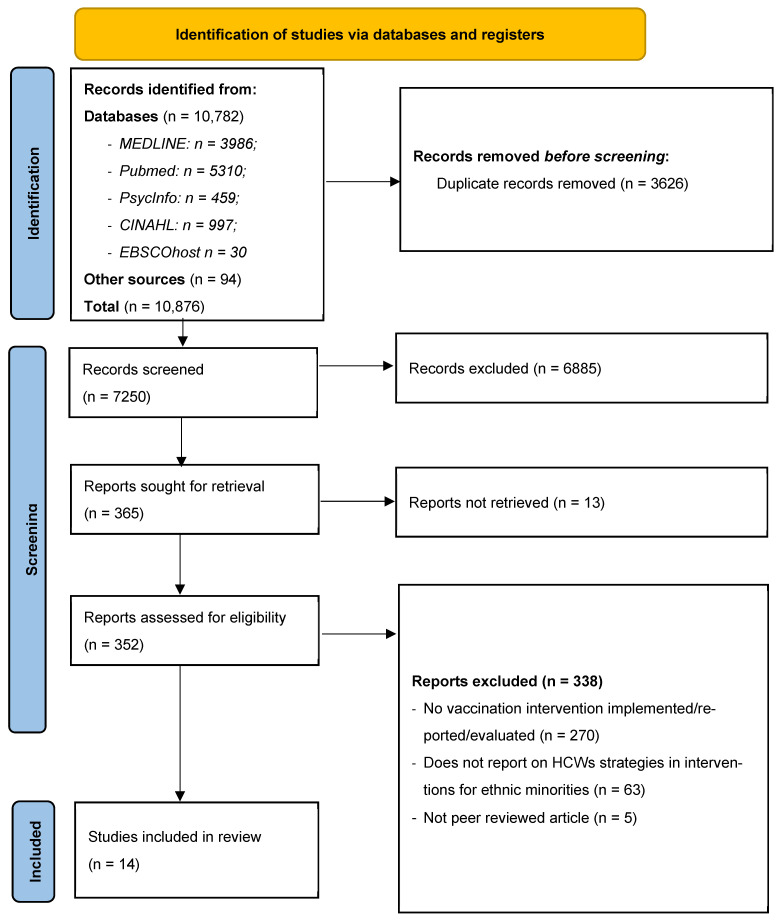
PRISMA flow chart outlining the selection of studies for the systematic review.

From the search, a total of 10,876 records were identified: 10,782 from database searches and 94 from hand searching other sources (specific journals on vaccination, preprints, and Google Scholar). Of these, 3626 duplicate records were removed before screening. Titles and abstracts for 7250 records were screened, of which 6885 were excluded, and 365 reports were sought for retrieval, but 13 reports were not available for retrieval. From this, 352 reports were assessed for eligibility at the full-text phase (see Figure 1). Following full-text review, 338 studies were excluded for reasons such as lacking relevant interventions, not including ethnic minority populations, lacking HCW activities, and not reporting any intervention outcomes. Four studies reported details on the same intervention and population and were merged and reported as one study [33,34,35,36]. In total, 14 studies were included in the review, based on 17 papers related to interventions targeting HCWs supporting ethnic minority communities. Articles retrieved during this process that did not relate to HCWs but did report interventions targeting ethnic minority groups were excluded from this review and reported as a separate review topic [37].

### 4.2. Study Characteristics

Table 2 provides a summary of intervention characteristics and results across the 14 included studies. Interventions were conducted in three countries: the United States (n = 12), the United Kingdom (n = 1) and New Zealand (n = 1). The studies included a total of 275,180 patients; however, several studies did not report the specific numbers of HCWs. All studies included intervention strategies implemented by HCWs to improve vaccination uptake and reduce hesitancy among ethnic minority populations. HCWs ethnicities were not reported but ethnic minority communities targeted by the interventions included Black/African American (n = 9 studies), Hispanic/Latino (n = 9), Asian (n = 6), Hawaiian/Pacific Islander (n = 3), Alaskan Native (n = 1), American Indian (n = 1), Māori (n = 1), and Russian (n = 1). Only one study reported interventions targeting vaccination uptake among HCWs, but the intervention did not specify the HCWs ethnicities [38].

Vaccines targeted by the interventions included a range of illnesses including influenza [38,39,41,45,47,48,50], pneumococcal [40,41,48], pertussis [33,35,41,42,46,50], tetanus [41,42], diphtheria [41,42], meningitis [42] and hepatitis B [43].

The most common study designs were cohort studies (n = 6) [33,38,39,40,43,46] and randomised controlled trials (RCTs) (n = 5) that were not blinded [41,44,47,48,49], of which one study did not report a comparison group [49] followed by cross-sectional studies (n = 3) [42,45,50], with two of these studies employing a mixed-methods cross-sectional design; Greenfield et al. [42] used a survey and focus group, and Skirrow et al. [50] evaluated routine data records and conducted interviews. Ten studies collected pre- and post-test data [33,34,35,36,39,40,42,43,44,46,47,48,49]. In addition to vaccine uptake, other outcomes reported included vaccine beliefs and perceptions, initiation and completion rates, missed opportunities, hesitancy, communication, and patient decision-making process.

Intervention settings were health centres (n = 11) [33,35,38,39,41,43,44,45,47,48,49,50], and community settings such as community pharmacies (n = 3) [42,46,49]. The interventions were conducted between 1995 and 2019 ranging from 2 months to 5 years (min = 2 months, max = 60 months, average = 17 months. HCWs included doctors, specialist physicians (e.g., obstetricians, physician directors), nurses, administrative staff and hospital interpreters.

### 4.3. Risk of Bias Results

The studies were of moderate to high quality and had a moderate risk of bias (moderate-quality = nine studies, high-quality = five studies) (Supplementary File S3). Potential risk of bias included insufficient detail about methods and results in some cross-sectional studies; unclear confounders and follow-up details in some cohort studies; unblinded participants in RCT studies, and limited qualitative methodological rigour in the mixed-methods study. These potential biases, in addition to the lack of ethnicity data for HCWs, implies caution needs to be taken when interpreting the findings, especially when assessing the results based on other sociodemographic factors.

### 4.4. Intervention Effects and Outcomes

The narrative synthesis grouped data based on COM-B constructs related to barriers and facilitators influencing vaccine uptake, BCW intervention types, policy options and BCTs. As shown in Table 3, outcomes of interest primarily focused on vaccine uptake and coverage amongst ethnic minority patients/participants [33,35,38,39,40,41,43,45,46,47,48,50]. While interventions targeting HCWs behaviour were designed to increase the confidence of physicians communicating about vaccines [44] and midwives providing vaccine recommendations to patients [50], other outcomes were related to patients’ behaviour change and included hesitancy [44] and missed vaccine uptake opportunities [49] in patients, not HCWs.

The physician-targeted communication intervention aimed to build HCW communication confidence and provide training on a novel communication strategy using an “Ask, Acknowledge, Advise” approach with parents [44]. At the end of the intervention, the reported HCW self-efficacy showed a significant improvement in establishing ongoing dialogue about vaccines with patients (Adjusted Odds Ratio (AOR) = 3.49, 95% CI 1.09–11.36, *p* = 0.03), but no statistically significant change in communication with parents about vaccines and in the talk about the benefits of vaccines [44]. Studies that used the improving HCWs recommendation approach targeted HCWs working with adolescents [42] and pharmacies delivering vaccinations [46]. The first intervention provided a presentation to HCWs that emphasised their distinctive role as the most trusted source of health information amongst ethnic minorities; hence, the need for HCWs to make strong vaccine recommendations using culturally appropriate messages and conversations. A follow-up survey showed that all HCWs that completed the survey post-presentation (n = 15 out 30 participating HCWs) reported routinely assessing immunisation status during well-child visits, 86% (n = 13) assessed during sports physicals, 52% (n = 8) assessed during acute-illness visits, and 33% (n = 5) assessed during injury visits [42]. The intervention strategies for pharmacies included training for pharmacists and vaccine promotion information for pharmacy staff and midwives, and the outcome post-intervention showed higher pertussis vaccine delivery in the intervention region compared to the control (26% vs. 0%). The intervention region also provided free vaccines compared to the control regions where women needed to pay for administration in the pharmacy [46].

### 4.5. Intervention Behaviour Change Components

Most interventions targeted psychological capability (n = 13 studies), reflective motivation (n = 12), and physical opportunity (n = 11) (Table 4) from the COM-B model. Seven of the nine BCW intervention types were identified: Education (n = 13 studies), Persuasion (n = 13), Enablement (n = 13), Training (n = 9), Environmental restructuring (n = 8), Incentivisation (n = 2) and Modelling (n = 1). The intervention types not identified were Coercion and Restriction. Six of the seven BCW policy options were observed: Communication/marketing (n = 12 studies), Service provision (n = 12), Environmental/social planning (n = 11), Guidelines (n = 7), Fiscal measures (n = 2), and Regulation (n = 2). One policy category was not identified (Legislation).

Twenty-two BCTs, from ten of the sixteen BCTTv1 groups, were identified across all studies (Table 5). The 10 BCT groups were: Goals and planning, Feedback and monitoring, Social support, Shaping knowledge, Natural consequences, Association, Comparison of outcomes, Reward and threat, Antecedents, and Identity. BCTs targeted HCWs behaviour directly and indirectly. An example of a direct behaviour change intervention strategy was the use of medical record prompts/cues to remind HCWs of the vaccination schedules of their patients [38,40,41,43,44,45,47,48,49]. An indirect strategy was HCWs providing patients with information resources to increase knowledge and support the decision-making process [42,45,48]. Details about the specific BCTs within each intervention are provided in Supplementary File S4.

The most commonly used BCTs were prompts/cues (n = 13 studies), credible source (n = 10), information about health consequences (n = 9), practical social support (n = 8), restructuring the physical environment (n = 7), adding objects to the environment (n = 6), instruction on how to perform the behaviour (n = 6), and monitoring of outcomes of behaviour without feedback (n = 5). All interventions included multiple BCTs ranging from ten [39] to three BCTs [42,43]. Across all studies, an average of six BCTs were used. The intervention by Nowalk et al. [48] was the most comprehensive, using a variety of culturally evidence-based approaches in different clinical settings and included standing orders (predetermined prescriptions exercised by HCWs when conditions have been met in patients), nursing visits, education, posters, videos, reminders, walk-in clinic, advertisement, competition, and incentives/rewards (free flu shot coupons and treats at the time of vaccination). Interventions conducted in a non-clinical setting used an educational approach with culturally specific brochures for the public and training for HCWs [42,46,49]. The use of prompts as a reminder was the most common approach targeted toward healthcare providers, and included vaccination reminders [38,39,40,41,43,44,45,47,48,49], acceptance or refusal stickers on patient charts [45,48], and pocket-sized cards listing recommended vaccine schedules [49].

### 4.6. Intervention Types and Policy Options

The effects of the different identified intervention types and associated BCTs varied.

#### 4.6.1. Education

Ten interventions included an education component targeting both HCWs and ethnic minority populations. This included education packages that contained vaccine information to be given directly to ethnic minority populations and delivered in local languages, e.g., into Apache language during the radio announcement for American Indian communities [33,35,38,42,45,48] and during direct interactions with HCWs during clinic visits delivering education about the benefits and risks of vaccines [33,35,39,40,44,46,47]. Most of the interventions to educate HCWs were trying to raise HCWs’ awareness of how to change the behaviour of their patient and community members. Education interventions appeared to increase vaccination coverage. For example, Hoppe & Eckert [45] provided educational sessions to all staff and showed influenza prevention videos to pregnant women in nine languages to increase their acceptance of the influenza vaccine. Healy et al. [33,35], who reported providers’ education for pertussis vaccine recommendation strategy for young infants through obstetric services, reported 72% (1129/1570) of postpartum women received Tdap prior to hospital discharge, and most of those had no contraindications (96.2%, 1129/1174). The unimmunised women (n = 441) were mostly from Hispanic (85.9%) and Black (10.2%) ethnicities, with more Black women refusing vaccines than other ethnicities (24% vs. 8%; *p* = 0.003). The education intervention components identified that all fall within the communication/marketing policy option.

#### 4.6.2. Persuasion

Different persuasion strategies were used by HCWs and these were often linked to education. These included the use of culturally specific information to persuade certain ethnic communities [38,42,48], providing a rationale for the intervention and displaying information in clinics [33,35,45] and having physicians invite patients to ask vaccine questions, share their concerns and make recommendations [40,41,43,44,47,49]. Studies did not report outcomes directly associated with the persuasion component. Henrikson et al. [44] reported improvement in provider–parent communication after the training intervention offered to physicians established an ongoing dialogue about vaccines, but this was not translated to high physician self-efficacy and confidence when talking about the benefits of vaccines. In another study [40], 21% of patients declined vaccination and 12% of patients continued to refuse vaccination despite recommendations from nurses and doctors, respectively, and this was mostly among Black patients compared to those from White and Asian ethnic backgrounds. Most of the persuasion techniques used were linked to the communication/marketing policy option.

#### 4.6.3. Enablement

Techniques to enable vaccine uptake were mostly cues, prompts, and standing orders which acted as proactive reminders for HCWs to screen and vaccinate patients. Enablement in the studies included communication strategies to guide HCWs on how to engage with patients [33,35,41,44] and the use of manual and electronic prompts or reminders used to tag patients files by vaccine status categorisation and updates which enabled HCWs to monitor or review vaccination status [38,39,40,41,43,44,45,47,48,49]. The policy options were service provision, environmental/social planning, and guidelines; these included interventions that delivered specific vaccine services to encourage vaccine access and uptake, especially nurse home visits [38,42,47,48], provided health providers with pocket-sized laminate cards listing the recommended vaccine schedule, contraindications, and minimum interval schedule [49], and use of certified medical interpreters with, in a typical month, 46% of visits interpreted, resulting in similar acceptance rates between English and non-English speaking patients (78% and 75%, respectively) [45]. This group of interventions appeared to be effective in increasing HCWs proactivity in vaccination service provision. However, Daniels et al. [40] reported that after the implementation of a standing order strategy, 21% of the adults surveyed declined vaccination following recommendations from nurses, but this reduced to 12% after consultations with a physician, indicating interactions with HCWs in addition to cues provided to physicians encouraged a change in behaviour among patients.

At the system-level, through practice-based database tracking and outreach implemented by Humiston et al. [47], significant improvement in vaccine coverage among Black/African American and Hispanic/Latino groups was observed (intervention vs. control, 64% vs. 22%; OR = 6.27; 95% CI = 5.42–7.2 *p* = 0.0001). The outcome indicated that patients who were tracked by HCWs with reminders of their patients vaccine status, and followed up during outreach activity, were six times more likely to receive influenza vaccines. The intervention was also effective across gender, ethnicity, age, and health insurance subgroups. However, ethnic immunisation rate disparities were not totally eliminated (*p* < 0.0001), and intervention group vaccine refusal (n = 1748) was highest among African Americans (3.5%) compared to other ethnicities and mostly among women vs. men (3.7% vs. 1.9%, *p* = 0.0236). From a community perspective, the intervention in the study by Nowalk et al. [48], which provided various culturally appropriate evidence-based interventions, reported inconsistent vaccination rate changes during the four years of the intervention. For instance, no significant difference in influenza vaccine uptake was observed between the intervention and non-intervention sites that compared the implementation of several culturally appropriate evidence-based interventions in Year 2 of the five years. Still, overall change over the four years was significant (pre-intervention rate 27.1% to 48.9% for intervention sites (*p* < 0.001) and 19.7% at control sites (*p* < 0.001)). The rates of PPV vaccination did not increase significantly overall for a group of patients aged 65 years and older. In addition, there was no improvement in uptake among ethnic groups or across gender.

In another study, Debroy et al. [41] implemented a modified electronic health record clinical reminder that bundled together three adult vaccination reminders, presented patient vaccination history and included talking points for providers to address vaccine hesitancy. Uptake of influenza and other vaccinations was slightly higher among patients who saw primary care teams in the intervention group than patients who saw teams in the control group (22.3% vs. 20.8%), although this was not statistically significant. The intervention did not reduce racial disparities in the vaccination rate (Control group vs. Intervention group vaccination rates, Black patients = 18%, 20%, White patients = 24%, 26%). There were persistently low vaccination rates among Black patients regardless of their provider’s assignment to the treatment.

#### 4.6.4. Training

Nine studies included training elements which targeted HCWs. Training approaches included both on-site and web-based presentations that highlighted the distinctive roles of HCWs as trusted sources of health information and provided them with specific messages to use when attending to ethnic minority communities [42,44]. The training content was not reported in most studies, but often included general training on vaccination as well as on very specific conditions such as H1N1 risk for pregnant women [45]. The effect of the training was mixed; for instance, Greenfield et al. [42] showed that despite the training provided to HCWs, only 52% of HCWs assessed patient’s immunisation status during acute-illness visits and 33% assessed during injury visits. On the other hand, the training intervention offered at 30 clinics by Henrikson et al. [44] only reached 67% (179/265) of eligible physicians. Physician self-efficacy in communicating was not significantly different between the intervention and control groups along with a non-significant difference in maternal vaccine hesitancy between the two groups at the end of the intervention.

#### 4.6.5. Environmental Restructuring

Eight studies restructured the vaccination delivery environment, and this is linked to the environmental/social planning policy option. Healy et al. [33,34,35,36] used a community approach to implement a cocooning strategy which provided vaccination to postpartum women before hospital discharge and also to their household and caregiver contacts. Other studies transformed part of the clinic to share information more passively such as an influenza prevention video continuously playing in the waiting room [45,48] and public health nurses providing home visits and vaccinations in addition to direct education [38,45] used prompts that displayed vaccine acceptance or refusal stickers on the front of patient charts, which in combination with other intervention elements, showed the overall immunisation within the first month of vaccine availability was 76%.

Environmental restructuring outside the health facilities showed mixed outcomes. For example, the cocooning strategy by Healy et al. [33,35] reported high vaccination rates, but this was not observed equally across different ethnicities; and the community-based intervention by Nowalk et al. [48] showed no significant improvement in increasing PPV vaccination rates. The vaccination intervention by Traeger et al. [38], which implemented community and home vaccination services and targeting vaccination for both HCWs and patients for a nationwide health facility, showed higher vaccination rates compared to the national average for HCWs (72.8% to national 36%) and people ≥65 years old (73.3% to national 51.4%). The intervention reduced the risk of a lower vaccination rate two-fold. This study also included staff and community education and vaccine standing orders (prompts), which have previously shown positive intervention effects.

#### 4.6.6. Incentivisation

Two studies included incentive provision as part of the intervention [43,48]. These strategies fall under the communication/marketing policy option. Hechter et al. [43] provided vaccines at no charge as an incentive for health provider members to receive immunizations within the system, while health providers outside the system were reimbursed by an agreed health plan. Nowalk et al. [48] targeted community members and mailed vaccine reminders with a ‘‘free flu shot coupon’’ to all eligible adults, sponsoring a contest for the most prolific vaccinator, and providing both vaccinators and vaccinated patients a treat at the time of vaccination. The intervention with a health provider incentive showed a significant increase in vaccine coverage rates compared to the control site, which had no reminders, and thus no incentive (annual vaccine initiation rates: RRR = 70.7, 95% CI: 62.8–79.6; third dose completion rates (RRR = 18.7, 95% CI: 14.2–24.8)). While the community incentivised intervention was effective, it did not have as much of an impact on ethnic groups or across different genders [48]. These findings indicate incentivisation to HCWs helped boost vaccination compared to the community-based intervention, which did not appear to have an impact in addressing ethnic differences in vaccination uptake.

### 4.7. Barriers and Facilitators Influencing Vaccine Uptake

#### 4.7.1. Barriers to Vaccine Uptake That Affect Ethnic Minority Communities

Eight studies reported barriers and predictors of vaccine hesitancy in ethnic minority communities [33,35,39,40,41,42,46,47,50] (Table 6). These included having underlying medical conditions [33,35], beliefs and misperceptions about the diseases [39,40,42,50], history of previous reactions to vaccines [33,35,39], religious objections [33,35], sourcing information from country of origin [42], poverty [33,35,46], poor patient access to preventive health services or health information [33,35,42,46], mistrust in the healthcare system [50], Black ethnicity [33,35,40,41,47,48] and HCW provider fatigue, which suggests that vaccination rates were significantly lower in patients seen later in the day than earlier in the day [41].

In addition to patients beliefs that vaccination was unnecessary and fear of vaccine-induced illness, there was a desire for more information regarding the vaccine [33,35,40,42]. Healy et al. [33] identified practical barriers to full cocooning (vaccinating postpartum women before hospital discharge and to household and caregiver contacts) which relates to physical opportunity in the BCW, such as limited vaccination hours, visiting restrictions due to the H1N1 pandemic ongoing at that period; and psychological capability factors such as inaccurate recall of vaccination history [33]. Greenfield et al. [42] reported several barriers to uptake of adolescent immunizations which included limited vaccine awareness and misperceptions (psychological capability), lack of physician recommendations (social opportunity) and the inability to access health information in native languages (psychological capability).

#### 4.7.2. Facilitators of Vaccine Uptake in Ethnic Minority Communities

Eight studies reported facilitators of vaccine uptake, such as access to verifiable vaccination records through lifespan immunisation registries (physical opportunity) [33] and availability of vaccine information in native languages (psychological capabilitfy) with accompanying physician recommendations (social opportunity) [42]. Physician recommendations and addressing patients’ vaccine-related concerns was also reported by Daniels et al. [40] and Traegar et al. [38]. Having positive interactions with healthcare workers, understanding the risk of the disease (psychological capability) and having trust in the wider public healthcare system (reflective motivation) can facilitate vaccine uptake [50]. Howe et al. [46] identified time to discuss vaccinations, overcoming transportation issues and other needs (physical opportunity) helped share vaccination messages; and funding vaccines with the support of GPs and subsidising training in community pharmacies can help increase uptake (physical opportunity). Having vaccination reminders that are intuitive and easy to use can reduce provider burden and fatigue and help increase vaccination rates (psychological capability) [41].

#### 4.7.3. Challenges of Intervention Implementation

Thirteen studies reported challenges faced during intervention implementation [33,35,39,40,41,42,43,44,45,46,47,48,49,50]. These include patient hesitancy factors (reported in Section 4.7.1), costs and time required for the intervention [33,35,43,44,47,48], and providers not making use of all the features within the intervention, emphasising the importance of making prompts more salient or providing further training in the use of talking points to address vaccine hesitancy [41]. Cost influenced participant selection as only a limited number of study participants could be recruited [44,48], individualised educational strategies needed for the cocooning intervention were expensive [33,35] and short intervention duration (6 to 12 months) did not allow sufficient time to observe statistically significant changes related to the intervention [43,44]. The use of a single clinic for one intervention means the results may not be generalisable to other settings [39,40,49]; inaccuracies in information translated to native languages [42] and poor records of vaccine availability and administration [44,48] meant a complete inventory of all vaccines received by different ethnic groups was not available as a complete measure of vaccine uptake in different settings (community vs. medical centre) [48]. Data challenges were reported in five studies [44,46,47,48,50] with limited availability of data resulting in likely underestimation of the number of vaccines administered.

External factors included influenza vaccine shortages [48] and increased media attention regarding H1N1 in 2009, which both encouraged and discouraged the use of immunisation, hence the study was unable to separate out the impact of this strong media attention versus the clinic efforts in achieving high coverage [45] and a new state law during the Henrikson et al. [44] study period which was highly publicised and may have altered vaccine hesitancy in the study population.

## 5. Discussion

This systematic review identified 14 interventions across three countries that reported interventions targeted at HCWs to increase vaccine uptake among ethnic minority populations, but none focused on ethnic minority HCWs. All the studies reported that the interventions were somewhat effective, with few showing only minimal improvement in vaccine uptake and/or reduction in hesitancy. Twenty-two BCTs with an average of six BCTs used, seven BCW intervention types and six policy options were identified to promote vaccine uptake. The most common BCTs were Prompts/cues (as a form of behavioural cueing, e.g., immunisation prompts in electronic medical records), Credible source (e.g., physicians inviting patients vaccine questions and concerns), Information about health consequences (e.g., to increase risk awareness and vaccine knowledge), Social support (practical) (e.g., nurses available to provide support and answer questions), Restructuring the physical environment (to change environmental context, e.g., vaccinations available in community pharmacies), Adding objects to the environment (to change environmental resources, e.g., providers given pocket-sized cards listing recommended vaccine schedules), Monitoring of outcome(s) of behaviour without feedback (e.g., electronic record of vaccine registry updated daily), and Instruction on how to perform the behaviour (vaccination information and instructions for targeted individuals). The BCTs that recurred across effective interventions included prompts/cues, credible source and information about health consequences which highlights the importance of the physical and social environment and provision of reminders and information within consultations to support HCWs to routinely promote vaccine uptake.

Based on the BCW, the intervention types that appeared most effective in improving vaccination rates were those that provided support to HCWs through training, education and enablement, and encouraged proactive engagement with communities. The behavioural mechanisms underpinning these intervention types include building knowledge and skills through training and education pathways which can address barriers relating to low vaccine knowledge; removing practical barriers with the provision of structural support that enables timely access to information and reduces logistical barriers, and building trust and credibility with communities. These intervention types were mostly related to three policy options: communication/marketing, environmental/social planning, and service provision. Barriers identified were related to physical and psychological capability and social and physical opportunity; this included low vaccine awareness, misperceptions about the vaccine-preventable diseases, limited vaccination hours, inaccurate vaccination history recall, lack of physician recommendations and inability to access health information in native languages. Facilitators of some interventions included access to verifiable vaccination records and availability of vaccine information in native languages with accompanying physician recommendations.

Overall, there is no substantial evidence to recommend any specific intervention over another intervention to address vaccine uptake among ethnic minority populations; this is due to the vast array of intervention strategies used in the limited number of included studies, and heterogeneity of BCTs and ethnic minority populations. However, this review does demonstrate that most interventions had multi-components targeting capability, opportunity and motivation which indicates effectiveness is not linked to a single BCT or intervention strategy, rather effectiveness is linked to a combination of different BCTs and intervention types. Increasing knowledge and skills in HCWs is important but behaviour change requires system-level enablement to translate the knowledge and skills into action. The results indicated health systems and service provision are important facilitators and should inform recommendations accordingly, as illustrated by Ekezie et al. [51], who identified improved communication and education, providing culturally appropriate and tailored vaccine information to targeted populations as strategies to improve primary healthcare services for ethnic minorities. Wider evidence also indicates education, training and improving vaccine information awareness about safety [52], and reminders for HCWs are effective tools to improve vaccine dialogue with various groups and in different settings [53,54]. HCWs are the most trusted advisor and influencers of vaccination decisions [55,56]; however, some HCWs feel ill-equipped to answer questions or engage in difficult conversations with those who are reluctant to be vaccinated [44]. Equipping HCWs with tools to communicate with their patients establishes a more trusting and constructive dialogue [57]. Despite HCW-focused interventions, disparities remain even when HCWs intervene. This is important as it highlights that HCWs are necessary but not sufficient without wider system-level support. Our review showed that prompts and proactive reminders as well as improvement in communication between HCWs and community members encouraged higher vaccine uptake.

Not all eligible HCWs in some studies were reached or participated in the intervention offered [44]. Engaging the majority of HCWs is crucial for effective implementation and long-term sustainability of the intervention, in terms of the absolute number, proportion and representativeness willing to participate in an intervention, as advised by the ‘Reach’ domain within the RE-AIM framework, which advocates for intervention “Reach, Effectiveness, Adoption, Implementation, and Maintenance” [58]. As shown in other studies, training HCWs increases their knowledge about vaccine efficacy, safety and adverse events, and helps build their confidence and willingness to recommend vaccines to others [59,60]. Strong recommendations from HCWs are significant motivators for vaccine uptake, and not informing patients of the need or reminding them when they are due for vaccines can contribute to missed vaccination opportunities [61,62,63]. At the population level, while knowledge and awareness-raising strategies are essential, knowledge alone is not enough, as indicated in studies that identified the importance of societal endorsement [59,64] and multi-component strategies [52] in addition to availability of information. Intervention challenges include high resource cost (financial cost and vaccine supply), which means strong support from government and healthcare organisations is necessary to institute tailored, culturally appropriate approaches [46].

Interventions included in this review, aimed at improving dialogue between HCWs and the population to increase the rate of HCW vaccine recommendation to patients, had varied effects on vaccine uptake. Other studies have shown that tailored messages, rather than general information, were more effective in increasing intentions to vaccinate [52,65]. In the current review, only a few studies tailored the intervention to the communities, and this was achieved through culturally specific resource materials (e.g., brochures) and communication in native languages. Written and graphic materials in the interventions had varied effects on vaccination rates; for instance, the use of vaccine information in brochures was effective when used with other components such as radio broadcasts and community and home vaccination services, but other studies have shown that vaccine information used alone, although it can increase patients confidence [66], does not always significantly improve immunisation attitudes [67]. In contrast, the use of videos (graphic representations) of vaccination risks and benefits had a stronger positive effect on vaccination acceptance, similar to other studies [68]. In terms of message framing, positive information about the importance of preventative measures contributed to increased acceptance. This may have been related to the corrective information provided, which has been shown in other studies to reduce misperceptions [69,70]. Another interesting finding was the effect of incentives, which improved coverage when provided to HCWs compared to when given to community members and health providers at a system level. Having a system that provided vaccines to member organisations at no charge improved vaccine disbarment compared to outside providers who needed to make claims for reimbursements, which was time consuming [43]. These incentivisation drivers and disparities need to be explored further, especially in community trust and mistrust of incentive meanings.

The COM-B construct ‘psychological capability’ was included in the highest number of interventions designed to increase HCWs knowledge and understanding of barriers and strategies to overcome vaccine hesitancy in ethnic minority communities. This is important as it facilitates dialogue that is more meaningful and relevant to specific communities and can increase trust in the information provided [10]. The COM-B construct that was least addressed by existing interventions was automatic motivation. Automatic motivation underpins habitual influences on behaviour and if interventions do not target this construct, there is a risk that despite HCWs understanding the need to recommend vaccines (psychological capability) and having the confidence in their ability to do this (reflective motivation), it may not become integrated into routine clinical practice as the conscious effort required to plan and execute this behaviour (psychological capability) may add to existing work pressures and result in habitual patterns of behaviour such as excluding vaccine-related discussions. Future research should explore the role of automatic motivation and the ‘behavioural regulation’ domain of psychological capability in interventions to support HCWs.

### 5.1. Limitations of the Review

This review may be subject to selection bias since it focuses on only respiratory and routinely recommended vaccines. Publication bias may also affect the study as only English language peer-reviewed articles were considered, ruling out research from non-English speaking countries that did not publish a potentially eligible study in English, and it may have missed unsuccessful interventions that are less likely to be documented in the peer-reviewed literature. Multi-component interventions were identified in some studies, but only overall impact data were presented; therefore, outcome data for individual strategies to address vaccine hesitancy within such studies were not separately available. Although only 14 studies were included and were mostly from the United States, the strength of the review shows the gap in existing interventions and strategies and the lack of global evidence. This emphasises the need for further work towards providing support to HCWs to improve vaccination coverage among ethnic minority groups.

### 5.2. Recommendations

The most effective interventions used multi-component strategies which were tailored to specific populations and addressed specific concerns, highlighting the importance of understanding the drivers of vaccine hesitancy to inform the specific content of intervention components (Table 7). Knowledge about vaccine importance (psychological capability) for both HCWs and ethnic minority populations, with tailored messaging that shows cultural appropriateness to social/professional role and identity (reflective motivation), was identified as a positive factor in the interventions. As a credible source, HCW encouragement and approval (social opportunity) led to increased vaccination acceptance. Incentives (that can enhance both reflective and automatic motivation) and reminders such as prompts and cues (automatic motivation) encouraged and supported HCWs to recommend vaccines and advise of vaccination appointments for their patients. Enhancing dialogue, cognitive and interpersonal skills of HCWs and ethnic minority populations (psychological capability) and positive message framing that overcomes negative emotions such as worry about side effects (reflective and automatic motivation) can increase the confidence of ethnic minority groups to accept the vaccination when eligible (reflective motivation).

To implement the recommendations in Table 7, there is a need to identify the hesitancy and concerns of ethnic minority populations through active listening to understand the reasons that underlie these concerns; this would help build trust [71]. Understanding the contextual factors (social, cultural, environmental and institutional determinants) to vaccination-specific issues would also be required to address the barriers for tailoring/personalising the messages to target population groups. Also, information needs to be evidenced with clear, positively framed messages (preferably in graphic formats) delivered through credible sources, such as HCWs, who should be supported on how to engage with ethnic minority populations [72]. This supports recommendations from a rapid review of public responses to messages encouraging vaccination against infectious diseases [73].

The influence of vaccination interventions by ethnicity, with consideration of HCW vaccination uptake (i.e., HCW personally receiving the vaccine) and their engagement in vaccine recommendations for ethnic minority groups (i.e., HCW hesitance in encouraging patient uptake) is not yet fully understood [74]. Although HCWs from ethnic minority groups can positively influence vaccination uptake, since they are also more likely to refuse vaccination, this reduces the number of trusted advisors and influencers within some communities. This highlights the importance of understanding the barriers and facilitators of vaccination uptake in HCWs from ethnic minority backgrounds.

## 6. Conclusions

This review extends our scientific understanding of the intervention strategies and components that promote positive vaccine delivery and uptake behaviour with a primary focus on supporting HCWs working with ethnic minority communities. Overall, this review found that, despite the extensive literature searching, only a few existing strategies are available to HCWs for addressing vaccine uptake among ethnic minority groups. Wide variation was observed in the intervention effects between studies, settings and target populations. The high heterogeneity across study outcomes limited our ability to draw general conclusions about the effectiveness of different strategies on specific population groups. Nonetheless, interventions to increase uptake that are multi-component and/or have a focus on dialogue-based approaches tend to perform better. The evidence on reminder prompts for HCWs showed positive effects and illustrated the potential to bring positive change by addressing the more practical aspects of vaccination. There is a clear need for more attention to understanding and addressing vaccine hesitancy and uptake at the community level. Some interventions identified in this review, even if mostly effective at increasing the capacity of the HCWs, did not show significant improvement in vaccine uptake among the ethnic minority populations, which highlights the importance of adopting a wider systems approach that extends beyond individual HCW-level interventions to reduce inequities in this area. Developing effective strategies to sustain trust in vaccination programmes requires an understanding of the particular social and psychological factors that determine the vaccination behaviours of different populations, and provision of resources and support for HCWs to facilitate this. This work offers novel insight that can facilitate the development of future vaccine initiatives globally, drawing from rigorous behavioural science methodology. Overall, interventions targeting multiple levels of influence in healthcare and community settings are likely yield better results than individual strategies and are necessary for all ethnic groups to achieve higher vaccination coverage.

## Figures and Tables

**Table 1:** Search terms used to identify relevant publications for the review.

Concept	Key Words
Vaccine hesitancy	vaccin* AND (hesitan* OR refus* OR confiden* OR accept* OR uptake* OR adopt*)
Ethnic minority groups	(ethnic group*) OR (ethnic minorit*) OR (minority group*) OR ethnic* OR minorit* OR race* OR racial OR Black* OR African* OR Asian* OR (South Asian*) OR Bangladeshi* OR (Pakistani*) OR Japanese OR Chinese OR Korean* OR Arab* OR BME OR BAME OR Roma* OR Hispanic* OR Caribbean* OR (people of color) OR (person of color) OR Jewish OR Jews OR gyps*
Intervention	Interven* OR communicat* OR train* OR motiv* OR strateg* OR guid* OR program* OR support* OR polic* OR approach* OR procedure* OR plan* OR engag*

**Table 2:** Description of vaccine interventions implemented by HCWs to support ethnic minority communities.

Study	Vaccine Focus	Study Design/Study Period/ (Intervention Duration)	Country(s) of Study	Reported Ethnic Minority Group	Setting and Patient Population Sample	HCW Type Sample (N)	Intervention/Comparison (Control)
Broderick et al. (2018) [39]	Influenza	Cohort (before and after intervention analysis) September 2014–September 2015 (12 months)	United States	Black Hispanic	University Medical Centre Patients: N = 228	Rheumatology providers (N not reported)	- Educational session - EMR alerts - Weekly email reminders
Daniels et al. (2006) [40]	Pneumococcal	Cohort 2004 (6 months)	United States	Asian Black/African American Latino Russian	General medical practice Patients: N = 370 adults	Nurses and Physicians (N = not reported)	- Standing orders - Physicians recommendations
Debroy et al. (2023) [41]	Influenza, Pneumococcal and diphtheria-pertussis-tetanus (DPT)	RCT October 2018–April 2019 (6 months)	United States	Black	Primary care Patients: Intervention group N = 13,934 (median age 60 years), control group N = 15,007 (median age 61 years)	Intake nurses and practitioners (N = not specified)	**Intervention:** - Modified EHR - Adult vaccination reminders - Talking points **Comparison:** - Reminders - Default EHR
Greenfield et al. (2015) [42]	Tetanus, Diphtheria, Pertussis, Meningitis, Human papillomavirus	Cross-sectional Mixed methods (survey, focus group) 2012/2013 (2 months)	United States	Black/African American Hispanic/Latino	Community Patients: N = 202 (157 parents, 45 adolescents)	HCW type (not specified) N = 30	- Brochure for public - HCW Training
Healy et al. (2009, 2011, 2012, 2015) [33,34,35,36]	Tetanus, Diphtheria, and Acellular Pertussis (Tdap) Infant Pertussis	Cohort January–April 2008 (4 months) June 2009–May 2011 (2 years)	United States	Asian Black Hispanic	Hospital Patients: N = 1571 postpartum women	Obstetricians and family practitioners, nurses, administrative staff and hospital interpreters (N = not reported)	- HCW training
Hechter et al. (2019) [43]	Hepatitis B	Cohort (Retrospective) 2012–2015 (12 months)	United States	Asian Black Hispanic Pacific Islander	Medical records Patients: Intervention group: N = 117,305 - Control group: N = 89,563 (19–59 years)	Physicians and nurses (N = not reported)	**Intervention:** - EMR reminders and alerts - Free vaccination **Comparison:** - Free vaccination only
Henrikson et al. (2015) [44]	General	RCT March 2012–December 2013 (6 months)	United States	Asian Black/African American Hispanic Pacific Islander	Hospital Patients: N = 347 mothers	Physicians (N = 179) Midlevel providers (N = 18) Nurses (N = 54) Other staff (N = 126)	**Intervention:** - Targeted communication - Prompts - Training **Comparison:** - No intervention components
Hoppe & Eckert (2011) [45]	Influenza	Cross-sectional (Retrospective) 2009 (Duration: NR)	United States	Hispanic	Medical Centre Patients: N = 158 pregnant women	Front desk staff, medical assistants, nurses, social workers, medical interpreters (N = not reported)	- Education - Prompts and reminders - Standing orders - Facilitated patient appointments and transportation
Howe et al. (2022) [46]	Pertussis	Cohort (before and after intervention analysis) 2015–2019 (4 years)	New Zealand	Asian Māori Pacific Islander	Community Patients: N = 27,576 eligible pregnant women in the maternity database	Pharmacists (N = not reported)	**Intervention:** - Education - Vaccine promotion **Comparison:** - No intervention
Humiston et al. (2011) [47]	Influenza	RCT 2003–2004 (Duration 4–5 months)	United States	Black/African American Hispanic/Latino	Primary care centres Patients: N = 3752	Outreach workers (N = not reported)	**Intervention:** - Practice-based database tracking/outreach - Provider prompts (system-level intervention) **Comparison:** - Routine care
Nowalk et al. (2008) [48]	Influenza Pneumococcal	RCT October 2001–November 2006 (5 years)	United States	Black/African American	Health centre Patients: N = 569 ≥50 years old	Clinical staff (N = not specified)	**Intervention:** - Standing orders, - EMR prompts - Education - Reminders - Incentives **Comparison:** - No intervention offered
Sabnis et al. (2003) [49]	General	RCT January 1995–December 1996 (2 years)	United States	Hispanic	Community health centre Patients: N = 696 children	Physicians, nurses (N = not specified)	- Education - Feedback - Prompts
Skirrow et al. 2020 [50]	Whooping cough (pertussis) Seasonal-influenza	Cross-sectional, Mixed-methods evaluation Electronic maternity records Interviews April 2017–March 2018 (12 months)	UK	Asian Black	Hospital Patients: N = Clinical data (n = 1710) Qualitative interview (n = 10 women)	Vaccine midwives (N = 2)	- Midwife-led clinic - Training on maternal vaccine delivery - Counselling
Traeger et al. (2006) [38]	Influenza	Cohort October 2002–April 2003 (6 months)	United States	American Indians Alaskan Natives	Hospital Patients: N = 2182 50–64 years (N = 1277), ≥60 years (N = 547)	Physicians (N = 18) Nurse practitioners (N = 5) Public health nurses (N = 10)	**Intervention:** - Education - Standing orders - Hospital vaccination - Community and home vaccinations **Comparison:** - Vaccination rates in other US populations

Note: EMR = Electronic Medical Record, EHR = Electronic Health Record.

**Table 3:** Vaccine interventions outcomes, direction of effect and study quality.

**Study**	**Intervention/Comparison (Control)**	**Intervention Outcomes**	**Vaccine Coverage/Uptake**	**Direction of Effectiveness**	**Study Quality** **(Score)**
Broderick et al. (2018) [39]	- Educational session - EMR alerts - Weekly email reminders	- Improved uptake vaccination in patients - Less delay in time to vaccination - >50% reduction in missed point-of-care vaccination opportunities	- Missed vaccination opportunities: reduced from 47% to 23%	↑ Increase in uptake ↓ Decrease missed opportunities	High (9/11)
Daniels et al. (2006) [40]	- Standing orders - Physicians recommendations	- 78 (21%) declined vaccination - 43(12%) continued to refuse vaccination - 327 (88%) patients accepted vaccination - Black (19%) more likely to decline compared with Whites (8%) and Asians (5%) (*p* = 0.01).	- Accepted vaccination: 88% (327/370).	↑ Increase in acceptance ↔ No clear change uptake in the Black community	Moderate (7/11)
Debroy et al. (2023) [41]	**Intervention:** - Modified EHR - Adult vaccination reminders - Talking points **Comparison:** - Reminders - Default EHR	- Uptake intervention vs. control group: (22.3% vs. 20.8%) - Low vaccination rates among Black patients - Patients seen later in the day less likely to receive an influenza vaccination (*p* < 0.001) - Time-of-day differences (*p* = 0.13)	- Intervention rates: Black patients = 20%, White patients = 26% - Control rates: Black patients = 18%, White patients = 24%	↔ No clear change	Moderate (10/13)
Greenfield et al. (2015) [42]	- Brochure for public - HCW Training	- HCPs (50%, 15/30) routinely recommended - 52% assessed patient’s immunisation status during acute-illness visits - 33% assessed during injury visits. - 7 HCPs reported not routinely recommending at least one of the three adolescent vaccines - 100% more likely to recommend HPV vaccine	NR	↑ Increase vaccine recommendation	Moderate (15/20)
Healy et al. (2009, 2011, 2012, 2015) [33,34,35,36]	- HCW training	- 96.2% Tdap uptake in women without self-reported contraindications. - 69% received Tdap vaccine following the birth of a prior infant - 29% immunised during routine visits - Mothers received Tdap vaccine prior to delivery: mean = 13.7 months (median = 13.4 months [range = 2.3–23.9 months]) - Infants born during post-intervention period: 93.7% (15/16 mothers) received vaccine postpartum. Mothers and family contacts of 2 of the 3 infants born during the cocooning period received vaccine after infant’s birth, with 30% and 80% completion. - Unimmunized (n = 441): Hispanic (85.9%), Black (10.2%), Asian (1.8%), White (1.1%), Other (0.5%). Black women refused Tdap more often than other ethnicities (24% vs. 8%; *p* = 0.003)	- 72% (1129/1570) received vaccine prior to hospital discharge. - 18% (19/105) received vaccine during current pregnancy.	↑ Increase in uptake	Moderate (7/11)
Hechter et al. (2019) [43]	**Intervention:** - EMR reminders and alerts - Free vaccination **Comparison:** - Free vaccination only	- Coverage increased at the intervention site, but low at the control site. - Changes in annual vaccine initiation rates (RRR = 70.7, 95% CI = 62.8–79.6); - Change in third dose completion rates (RRR = 18.7, 95% CI = 14.2–24.8).	- Patients who received at least one dose of Hep B vaccine: Intervention site-(12.3% and 66.6%), Control (14.5% and 16.8%). - 3-dose series coverage rate (years 2012 and 2015): Intervention site (7.6% and 29.4%), control site (9.4% and 10.8%).	↑ Increase in uptake	High (11/11)
Henrikson et al. (2015) [44]	**Intervention:** - Targeted communication - Prompts - Training **Comparison:** No intervention components	- Intervention training at 30 clinics reached 67% (179/265) physicians and 198 other staff (18 midlevel providers, 54 nurses, and 126 other staff). - Physician communication self-efficacy not significantly different between groups. - Change in maternal vaccine hesitancy: Intervention (9.8% to 7.5%), control (12.6% to 8.0%) (*p* = 0.78). No detectable effect of intervention (AOR) = 1.22, 95% CI = 0.47–2.68). - Physician Self-efficacy: Communicate with parents about vaccines (AOR = 1.17, 95% CI = 0.49–2.93, *p* = 0.57). Establish an ongoing dialogue about vaccines (AOR = 3.49, 95% CI = 1.09–11.36, *p* = 0.03), Talk about the benefits of vaccines (AOR = 1.88, 95% CI = 0.55–6.47, *p* = 0.61).	NR	↔ No clear change in physician communication self-efficacy ↔ No clear change in vaccine hesitancy	High (11/13)
Hoppe & Eckert (2011) [45]	- Education - Prompts and reminders - Standing orders - Facilitated patient appointments and transportation	- Increase in acceptance rates: Vaccine acceptance = African American (71%, 27/38), West/East African (74%, 52/70), Pacific Islander/Asian (78%, 7/9), Hispanic (81%, 13/16), Caucasian (90%, 17/19), Native American (100%, 2/2). 46% of visits interpreted visits. - Vaccine acceptance: English speaking patients (78%, 59/76), non-English speaking patients (75%, 59/79). No statistical difference in H1N1 vaccine acceptance rate (*p* = 0.667).	- Immunised within first month of vaccine availability: 76% (120/157). - Acceptance rates by ethnicity: African American (71%, 27/38), West/East African (74%, 52/70), Pacific Islander/Asian (78%, 7/9), Native American (100%, 2/2), Caucasian (90%, 17/19), Hispanic (81%, 13/16). - No difference in acceptance rate (*p* = 0.667).	↑ Increase in acceptance ↔ No clear change in H1NI vaccine acceptance	Moderate (14/20)
Howe et al. (2022) [46]	**Intervention:** - Education - Vaccine promotion **Comparison:** No intervention	- Maternal pertussis (%pre- to post-intervention; %change): intervention (21% to 35%; 67%), control (26% to 38%; 44%), (*p* = 0.0014). - Māori women (post- vs. pre-intervention): intervention (117%; OR = 2.42, 95% CI = 1.99–2.97), control (38%; OR = 1.50, 95% CI = 1.29–1.74) (*p* = 0.0281) - Non-Māori women (post- vs. pre-intervention): intervention (OR = 1.98, 95% CI = 1.58–2.03), control (OR = 1.78, 95% CI = 1.58–2.03) no significant difference. - Community pharmacies providing vaccinations: intervention region (42%, 35/83), control regions (38%, 28/73). - Urban areas more likely to offer vaccination services than pharmacies outside those areas: intervention region (60% vs. 21%), control regions (48% vs. 17%). - Pharmacies delivered pertussis vaccinations in post-intervention period: intervention region (26%, n = 1084), control region (0%, n = 7).	- Māori women: Pre-intervention (Control vs. Intervention—17.5% vs. 9.4%); post intervention (Control vs. Intervention—24.2% vs. 20.4%). - Non-Māori women: Pre-intervention (Control vs. Intervention—36.4% vs. 27.4%); post intervention (Control vs. Intervention—50.4.0% vs. 42.7%).	↑ Increase in uptake ↑ Increase in community pharmacy vaccinations ↑ Increase in urban vaccinations	High (11/11)
Humiston et al. (2011) [47]	- Practice-based database tracking/outreach - Provider prompts (system-level intervention) **Comparison:** Routine care	- Immunisation rates: intervention vs. control group (64% vs. 22%, *p* < 0.0001). Intervention group more likely to receive influenza vaccine (OR = 6.27; 95% CI = 5.42–7.26; *p* < 0.0001). Intervention effective across gender, race/ethnicity, age, and insurance subgroups. - Immunisation rate disparities: race/ethnicity (*p* < 0.0001) and insurance (*p* = 0.0005) group disparities were not overcome in the single influenza season intervention period. - Intervention group vaccine refusal (n = 1748): Ethnicity [African Americans 3.5%, White 3.2%, Hispanic 0.6%, and other races 1.7% (*p* = 0.18)], Gender [Females vs. males (3.7% vs. 1.9%, *p* = 0.0236)]. Hispanic seniors had lowest immunisation rate.	- Immunisation rates: intervention vs. control (64% vs. 22%, *p* = 0.0001).	↑ Increase in uptake	High (11/13)
Nowalk et al. (2008) [48]	**Intervention:** - Standing orders, - EMR prompts - Education - Reminders - Incentives **Comparison:** No intervention offered	- Influenza vaccination rates differed significantly between intervention and non-intervention sites overall in Year 1 (2001/02), Year 3 (2003/04), and Year 4 (2005/06), with no significant difference between intervention and non-intervention sites in Year 2 (2002/03). - Comparison of intervention and non-intervention sites: rates of PPV vaccination did not increase significantly for any year of the study for the overall group of patients ≥65 years old or either racial group. No ethnic/racial disparities observed. - Change in influenza vaccination rate: Pre-intervention rate of 27.1% increased to 48.9% for intervention sites (*p* < 0.001), concurrent control rate remained at 19.7% (*p* < 0.001). - Change in PPV rate among ≥65 years old: Pre-intervention rate of 48.3% to 81.3% (*p* < 0.001) in intervention site, 66.7% at control (concurrent) (*p* = 0.08). - Influenza vaccination association: with intervention (OR = 2.07, 95% CI = 1.77–2.41, *p* < 0.001), ≥65 years old (OR = 2.0, 95% CI = 1.62–2.48, *p* < 0.001), ethnicity (OR = 1.06, 95% CI = 0.83–1.34), gender (OR = 0.87, 95% CI = 0.69–1.10). - PPV vaccination association: with intervention (OR = 1.21, 95% CI = 0.99–1.47), ≥65 years old (OR = 1.10, 95% CI = 1.05–1.15, *p* < 0.001), ethnicity (OR = 1.15, 95% CI = 0.66–1.98), gender (OR = 0.98, 95% CI = 0.57–1.68).	- Pre-intervention rate of 27.1% increased to 48.9% for intervention sites (*p* < 0.001), concurrent control rate remained at 19.7% (*p* < 0.001).	↑ Increase in uptake overall ↔ No clear change in some years ↔ No clear change in ≥65 years old or any racial group	Moderate (10/13)
Sabnis et al. (2003) [49]	- Education - Feedback - Prompts	- Change in missed opportunities: 49% (173/352) to 13% (45/344), (*p* < 0.001). - Reasons for missed opportunities (n = 45): parent refusal (15.6%), moderate or severe illness (15.6%), incorrect documentation as “up-to-date” (13.3%). 28.9% missed opportunity for simultaneous immunisation. 26.6% with no reasons documented for missed opportunity visits.	NR	↓ Decrease missed opportunities	Moderate (8/13)
Skirrow et al. 2020 [50]	- Midwife-led clinic - Training on maternal vaccine delivery - Counselling	- 1710 women seen in clinic, 1429 vaccines administered, 281 vaccinations declined. - 3147 women delivered at the hospital site, 48% (1501/3147) seen in clinic, 89% (1334/1501) received one or two maternal vaccines. - 42% (1334/3147) of women delivering at the hospital received one or more maternal vaccines via the vaccine clinic; 4% (56/1334), received counselling prior to the vaccination date - 33%, ethnicity was not recorded - 4% (n = 63) of women seen in the clinic recorded not understanding English, of which 11% declined vaccination from the clinic.	- 83.6% (1429/1710) women seen in clinic vaccinated. - 51% (360/713) women vaccinated against flu at antenatal vaccine service. - 15% (n = 43) received pertussis and flu vaccines at separate appointments. - 40 women vaccinated with pertussis vaccine also received flu vaccine later in pregnancy - 11% (n = 75) received neither pertussis vaccine nor seasonal flu vaccine.	↑ Increase in uptake	Moderate (6/9)
Traeger et al. (2006) [38]	**Intervention:** - Education - Standing orders - Hospital vaccination - Community and home vaccinations **Comparison:** - Vaccination rates in other US populations	- Employees vaccinated (n = 375): HCWs (72.8%, n = 273), more than double 2001 national coverage (36%). - ≥65 years old vaccinated: 73.3%, 355/484, compared to national (51.2%, 21,578/42,110) with risk ratio for vaccination, RR = 2.18; 95% CI = 2.06–2.30, *p* < 0.0001).	- General vaccination coverage: ≥65 years and older (71.8%, 393/547), 50–64 years (49.6%, 633/1277), those with diabetes (70.2%, 766/1091). - WRSU employee vaccination rate: 72.8% (273/375).	↑ Increase in uptake among employees ↑ Increase in uptake in ≥65 years old	Moderate (7/11)

NOTE: AOR = Adjusted Odds Ratio, NR = Not report, RR = Relative risk.

**Table 4:** Summary of BCW components identified in interventions to support the promotion of vaccine uptake.

Study (Author, Year)	Constructs			Influences on Behaviour—COM-B						Intervention Types									Policy Options						
	Capability	Opportunity	Motivation	Physical Capability	Psychological Capability	Physical Opportunity	Social Opportunity	Reflective Motivation	Automatic Motivation	Education	Persuasion	Incentivisation	Coercion	Training	Restriction	Environmental Restructuring	Modelling	Enablement	Communication/Marketing	Guidelines	Fiscal Measures	Regulation	Legislation	Environmental/ Social Planning	Service Provision
Broderick et al. (2018) [39]	✓	✓	✓		✓	✓	✓	✓	✓	✓	✓			✓		✓	✓	✓	✓	✓				✓	✓
Daniels et al. (2006) [40]					✓	✓		✓		✓	✓					✓		✓	✓			✓		✓	
Debroy et al. (2023) [41]	✓	✓	✓		✓		✓	✓		✓	✓							✓		✓					✓
Greenfield et al. (2015) [42]	✓	✓	✓		✓	✓		✓		✓	✓			✓				✓	✓						✓
Healy et al. (2009, 2011, 2012, 2015) [33,34,35,36]	✓	✓	✓		✓	✓	✓	✓		✓	✓					✓		✓	✓	✓				✓	✓
Hechter et al. (2019) [43]					✓		✓				✓	✓						✓			✓	✓			
Henrikson et al. (2015) [44]	✓	✓	✓	✓	✓	✓	✓	✓		✓	✓			✓				✓	✓					✓	✓
Hoppe & Eckert (2011) [45]	✓	✓	✓	✓	✓		✓	✓	✓	✓	✓			✓		✓		✓	✓	✓				✓	✓
Howe et al. (2022) [46]	✓	✓	✓	✓	✓	✓	✓	✓		✓	✓					✓			✓		✓			✓	✓
Humiston et al. (2011) [47]	✓	✓	✓	✓	✓	✓		✓	✓	✓	✓			✓				✓	✓	✓				✓	✓
Nowalk et al. (2008) [48]	✓	✓	✓	✓	✓	✓	✓	✓		✓	✓	✓		✓		✓		✓	✓					✓	✓
Sabnis et al. (2003) [49]	✓	✓		✓	✓	✓				✓	✓			✓				✓	✓	✓				✓	✓
Skirrow et al. (2020) [50]		✓	✓			✓	✓	✓		✓				✓		✓		✓	✓					✓	✓
Traeger et al. (2006) [38]	✓	✓	✓	✓	✓	✓	✓	✓		✓	✓			✓		✓		✓	✓	✓				✓	✓
TOTAL (n = studies)	11	12	11	7	13	11	10	12	3	13	13	2	0	9	0	8	1	13	12	7	2	2	0	11	12

**Table 5:** Summary of BCTs identified in the vaccine interventions.

BCT Group	Identified BCTs within the Included Studies	Broderick et al. (2018) [39]	Daniels et al. (2006) [40]	Debroy et al. (2023) [41]	Greenfield et al. (2015) [42]	Healy et al. (2009, 2011, 2012, 2015) [33,34,35,36]	Hechter et al. (2019) [43]	Henrikson et al. (2015) [44]	Hoppe & Eckert (2011) [45]	Howe et al. 2022 [46]	Humiston et al. (2011) [47]	Nowalk et al. (2008) [48]	Sabnis et al. (2003) [49]	Skirrow et al. (2020) [50]	Traeger et al. (2006) [38]
1. Goals and planning	1.4. Action planning							✓							
2. Feedback and monitoring	2.1. Monitoring of behaviour by others without feedback		✓	✓					✓						
	2.2. Feedback on behaviour	✓											✓		
	2.4. Self-monitoring of outcome(s) of behaviour					✓									
	2.5. Monitoring of outcome(s) of behaviour without feedback			✓					✓		✓	✓			✓
	2.6 Biofeedback/performance monitoring by others	✓													
	2.7 Feedback on outcomes of behaviour	✓													
3. Social support	3.2. Social support (practical)	✓		✓		✓		✓	✓		✓			✓	✓
4. Shaping knowledge	4.1. Instruction on how to perform the behaviour	✓		✓				✓		✓			✓		✓
	4.2. Information about Antecedents					✓									✓
5. Natural consequences	5.1. Information about health consequences	✓			✓	✓		✓	✓		✓	✓	✓		✓
	5.2. Salience of consequences								✓						
7. Association	7.1. Prompts/cues	✓	✓	✓		✓	✓	✓	✓	✓	✓	✓	✓	✓	✓
9. Comparison of outcomes	9.1. Credible source	✓	✓	✓	✓	✓		✓		✓		✓		✓	✓
	9.2. Pros and cons							✓							
10. Reward and threat	10.1. Material incentive (behaviour)											✓			
	10.10. Reward (outcome)											✓			
	10.2. Material reward (behaviour)						✓					✓			
	10.8. Incentive (outcome)						✓					✓			
12. Antecedents	12.1. Restructuring the physical environment	✓						✓	✓		✓	✓		✓	✓
	12.5. Adding objects to the environment		✓						✓	✓	✓		✓		✓
13. Identity	13.2. Framing/reframing	✓		✓	✓										
	No. of BCTs within each study	10	4	7	3	6	3	8	8	4	6	9	5	4	9

**Table 6:** Factors influencing vaccine uptake and intervention implementations and recommendations.

Study	Hesitancy Predictors/Factors	Barriers to Uptake	Facilitators to Uptake	Intervention Challenges
Broderick et al. (2018) [39]	Younger age, less frequent rheumatology office visits, and higher ESR (erythrocyte sedimentation rate), negative attitudes toward vaccine safety and efficacy predicted missed opportunities pre-intervention. Post-intervention no decrease in missed opportunities in the non-Hispanic Black ethnicity group was observed. Residence outside the New York City metropolitan area, and non-English primary language associated with poorer uptake. History of previous adverse reaction to vaccines markedly reduced likelihood of vaccination.	Limited clinic time and competing priorities. Fragmented delivery and documentation systems. Infrequent clinic attendance. Incomplete or inaccurate vaccination records.	Active provider engagement in recommending vaccination. Weekly provider-specific email reminders.	Single-site academic design limits generalizability; lack of control group restricts causal inference. Reliance on patient recall for vaccinations received elsewhere introduces potential misclassification.
Daniels et al. (2006) [40]	Reasons for refusal: believing vaccination was unnecessary (32%), fearing shots in general (21%), fearing vaccine-induced illness (26%), wanting more vaccine information (9%). More racial and ethnic minorities than White people refused pneumococcal vaccination, particularly African Americans, because of negative attitudes and misconceptions about vaccination and because of fear of shots or vaccine-related illness.	NR	Using standing orders for nurses in primary care clinics to assess and offer vaccination to patients. Physician recommendation. Addressing patients’ vaccine-related concerns.	Study setting was a single general internal medicine clinic and results may not be generalizable to other settings.
Debroy et al. (2023) [41]	Treatment teams with higher concentrations of male, older, and rural patients have higher vaccination rates, while teams with higher concentrations of Black patients have lower vaccination rates.	Provider fatigue means that patients seen later in the day may be less likely to receive a vaccination.	Having a reminder that is intuitive and easy to use.	Providers may not make use of features within the intervention such as talking points of presumptive language. Future research should explore ways to make these prompts more salient or explicitly train providers in the delivery of this messaging.
Greenfield et al. (2015) [42]	Beliefs and misperceptions about the diseases. Information from country of origin.	Lack of physician recommendations. Inability to access health information in native languages.	Information in native language. Physician recommendation.	Native-language-speaking CHPs (Community Health Promotors) reviewed the professional commercial translations and found significant inaccuracies requiring revision of the materials.
Healy et al. (2009, 2011) [33,35]	Sociodemographic factors: Black ethnicity, older age group are less likely to be vaccinated. Other factors: History of previous reactions to vaccines, current illnesses, underlying medical conditions, religious objections, uncertainty of past vaccination, baby given up for adoption, poor access to preventive health services, poverty.	Limited vaccination hours. Visiting restrictions due to pandemic. Inaccurate recall of vaccination history.	Access to verifiable vaccination records. Extended vaccination hours.	Reasons for failure of Tdap vaccine administration: having previous Tdap, Td in pregnancy, Td within prior 2 years, medication for anaphylaxis, mother refused, no documented reasons. Maternal knowledge and recall of type of vaccine administration is unreliable, regardless of maternal age. Programme requires significant investment of resources. - Individualised educational strategies are costly and time-consuming. Tdap vaccine could have been administered to mothers and other family contacts of infants born at hospitals other than the intervention site, limiting the ability to assess the impact of the programme.
Hechter et al. (2019) [43]	NR	NR	NR	Decrease in vaccination rate after a few months following the initial increase suggested intervention effect may have waned over time. Longer observation period needed to evaluate the long-term intervention effect on vaccination rates.
Henrikson et al. (2015) [44]	NR	NR	NR	Null effect observed may have been due to: Intervention reach: do not know how many intervention physicians used the online training or whether physicians attended only partial in-person training so mothers could have encountered an “untrained” physician. Insufficient intervention intensity: 1-dose, 45 min training. Given the null effect on maternal vaccine hesitancy, study has limited ability to assess where the intervention may have fallen short in its implementation. During the study period, a highly publicised pertussis epidemic that involved infant deaths and a new state law requiring a healthcare provider’s signature to claim vaccine exemption for school entry may have altered vaccine hesitancy in the study population. Baseline differences in the racial and ethnic distribution of mothers, perhaps related to use of block randomization of clinics.
Hoppe & Eckert (2011) [45]	NR	NR	NR	Increased media attention regarding H1N1 in 2009 both encouraged and discouraged the use of immunisation.
Howe et al. (2022) [46]	NR	Barriers to maternal vaccination uptake included set-up costs, general practice negativity concerns, and insufficient qualified staff. Barriers affect pharmacies in high deprivation areas without a private market. Rural pharmacies which have greatest need given staffing shortages of nurses in rural general practice, and barriers for pregnant women living in poverty. Many pharmacies did not offer vaccinations; non-vaccinating pharmacy staff may not be sufficiently informed or proactive with maternal vaccinations, and some pharmacies did not offer vaccinations at all opening hours.	Ensuring early engagement with a Lead Maternity Carer allow time to discuss maternal vaccinations, overcoming transport issues and other urgent social needs likely to help maternal vaccination messaging to be received and sufficient time to get the vaccinations. Effect of promotional campaign indicates multi-pronged approach, including extending funding of maternal pertussis vaccination to community pharmacies, can help increase uptake.	Some vaccination administrations might still have been missed. Some vaccinations at hospitals may have been uncaptured by clinic data. The manual process for the Tdap pharmacy claims data resulted in some keying errors and input of claims dates not administration dates, leading to a likely loss of valid data matching the maternal database dates. Therefore, the number of vaccinations delivered in pharmacy to eligible women, and the effect of the intervention for pertussis vaccination is probably understated.
Humiston et al. (2011) [47]	The following groups had a higher refusal rate: Ethnicity—African American Age—Older (including White seniors) Gender—Female	NR	NR	Many primary care centres had substantial financial challenges as they served vulnerable populations. Provider reminders placed on patients charts in intervention group and knowledge about the study may have prompted greater vaccination of control subjects than would otherwise have occurred, leading to an underestimate of intervention effect (i.e., a conservative bias). Providers may have been less likely to check the patient’s immunisation record because they assumed that the absence of a reminder indicated prior vaccination. Some data on influenza vaccinations given in public clinics were not available.
Nowalk et al. (2008) [48]		NR	NR	Influenza vaccine delays or shortages occurred during study period in 2000/01 and 2004/05. Due to budget constraints, random sample of almost 600 patients chosen to represent patient population in thousands. Record review did not capture vaccines given in community settings such as workplaces, grocery stores, and pharmacies. Complete inventory of all vaccines received was not available. Included only patients enrolled in the clinics, those who left the clinic and recently coming not included.
Sabnis et al. (2003) [49]	NR	NR	NR	Study took place in a single clinical setting, limiting whether results can be generalised to other clinics.
Skirrow et al. (2020) [50]	Not believing in vaccines or vaccines in pregnancy. Perceived risks of the vaccine or the disease itself. Others perceived the risks of the vaccine to be too high and they declined vaccination. Mistrust in healthcare system. Black Afro Caribbean women were less likely to receive vaccine.	NR	Positive interactions with healthcare workers. Understanding the risk of disease. Trust in wider public healthcare system.	Number of women vaccinated in primary care during the same time-period unknown because of no access to GP records of the women attending the clinic or who delivered at the hospital. Therefore, study could not provide total vaccine uptake estimates for the whole hospital population. Can only report on the number of women who received or declined vaccines from the antenatal clinic. Some limitations from the use of routine data include ethnicity not recorded in a third of the women; which may have led to over or underestimation of the association between ethnicity and vaccine receipt.
Traeger et al. (2006) [38]	NR	NR	Standing orders greatly facilitated immunisation in clinics. Clinicians exploring patients’ reasons for vaccination refusals.	NR

**Table 7:** Recommendations of BCTs to use in interventions to support HCWs promoting vaccination uptake with ethnic minority groups.

BCT	Recommendations Suggested from the Implemented Intervention Experiences
Action planning	- Encourage physicians to plan towards giving strong recommendations to patients. - Additional effort and long-term initiatives are needed to prevent the intervention effect from waning (decrease in vaccination rate) over time.
Monitoring of outcome(s) of behaviour without feedback	- Use patient tracking/recall/outreach and provider prompts strategies to increase seasonal influenza immunisation rates.
Prompt/cues	- Standing order protocol (approved protocols that authorise appropriately trained HCWs to assess patient immunisation status and administer vaccinations without a written order or examination) can ensure wider vaccine offer. - Present HCWs more opportunities to vaccinate, such as allowing nurses to identify and tag medical charts of those needing immunisations. - Physicians can promote vaccination with regular reminders.
Credible source	- Reinforce interventions by trusted members of the community to improve immunisation rate. - Intervention sites should carry out outreach work in neighbourhoods to develop trust among community members.
Instruction on how to perform the behaviour	- Enhance ability to communicate effectively with non-English speaking patients, this will contribute to equivalent vaccine coverage and acceptance across all ethnic populations. - Native-language speakers should carefully review all translated materials before distribution, to validate language used and meaning. - Educate nurses and translators on the intervention prevention strategy rationale and allow adequate time and opportunity to address concerns. - Physicians respond well to intervention and information delivered in lecture style but change to improve HCW communication behaviour with patients may require higher training intensity and activities. - Improve provider knowledge of vaccinations and screening vaccine status at each visit to decrease missed opportunities. - Provide vaccination education and information forums to HCWs who answer queries or concerns, and HCWs can become powerful vaccine advocates
Social support (practical)	- Contacting patients personally in their own language to encourage clinic attendance and immunisation - Offering taxi transportation for women at or beyond 36 weeks gestation to clinics for vaccination - Specific culturally appropriate messages for tailoring conversations to address vaccine beliefs and misperceptions
Restructuring the physical environment	- Use standing orders and provision of vaccinations on-site to improve cocooning acceptance and success in high-risk population. - Interventions can be individualised to the culture, operational systems, staffing patterns, and patient populations of health centres. - Promote vaccines in school-based health settings to increase adolescent immunisation rates, since that is where adolescents spend a significant amount of time, thereby minimising scheduling and transportation barriers.

## Data Availability

No new data were created or analysed in this study. Data sharing is not applicable to this article.

## Supplementary Materials

The following supporting information can be downloaded at: https://www.mdpi.com/article/10.3390/healthcare14060749/s1, Supplementary File S1: Behaviour Change Technique Taxonomy v1 (BCTTv1); Supplementary File S2: OVID Medline Search Strategy; Supplementary File S3: Quality Assessment (Risk of Bias) of included studies; Supplementary File S4: Identified intervention components mapped to relevant BCTs. PRISMA 2020 abstract, checklist.

## Abbreviations

The following abbreviations are used in this manuscript:

ANC	Antenatal care
AOR	Adjusted Odds Ratio
BCT	Behaviour Change Technique
BCW	Behaviour Change Wheel
CI	Confidence Interval
GP	General Practitioner
HCP	Healthcare Provider/Practitioner
HCW	Healthcare Worker
NR	Not reported
OR	Odds Ratio
PPV	Pneumococcal polysaccharide vaccine
RR	Relative Risk
RRR	Ratio of the rate ratio
Tdap	Tetanus, Diphtheria, and Pertussis combination vaccine
UTD	Up to date
