# Supplementary material for: Intervention Strategies for Healthcare Workers to Promote Vaccine Uptake in Ethnic Minority Populations: A Systematic Review of Behaviour Change Techniques
Source: Healthcare (Basel). 2026 Mar 16;14(6):749. doi: 10.3390/healthcare14060749 (PMC13026132; doi:10.3390/healthcare14060749)
Supplement: Supplementary file 1 [file healthcare-14-00749-s001.zip › healthcare-4103327-Supplementary Files S1-S4.pdf]

Supplementary File S1: Behaviour Change Technique Taxonomy v1 (BCTTv1)

Page	Grouping and BCTs
1	**1. Goals and planning**<br>1.1. Goal setting (behavior)<br>1.2. Problem solving<br>1.3. Goal setting (outcome)<br>1.4. Action planning<br>1.5. Review behavior goal(s)<br>1.6. Discrepancy between current behavior and goal<br>1.7. Review outcome goal(s)<br>1.8. Behavioral contract<br>1.9. Commitment
3	**2. Feedback and monitoring**<br>2.1. Monitoring of behavior by others without feedback<br>2.2. Feedback on behaviour<br>2.3. Self-monitoring of behaviour<br>2.4. Self-monitoring of outcome(s) of behaviour<br>2.5. Monitoring of outcome(s) of behavior without feedback<br>2.6. Biofeedback<br>2.7. Feedback on outcome(s) of behavior
5	**3. Social support**<br>3.1. Social support (unspecified)<br>3.2. Social support (practical)<br>3.3. Social support (emotional)
6	**4. Shaping knowledge**<br>4.1. Instruction on how to perform the behavior<br>4.2. Information about Antecedents<br>4.3. Re-attribution<br>4.4. Behavioral experiments
7	**5. Natural consequences**<br>5.1. Information about health consequences<br>5.2. Salience of consequences<br>5.3. Information about social and environmental consequences<br>5.4. Monitoring of emotional consequences<br>5.5. Anticipated regret<br>5.6. Information about emotional consequences
8	**6. Comparison of behaviour**<br>6.1. Demonstration of the behavior<br>6.2. Social comparison<br>6.3. Information about others' approval
9	**7. Associations**<br>7.1. Prompts/cues<br>7.2. Cue signalling reward<br>7.3. Reduce prompts/cues<br>7.4. Remove access to the reward<br>7.5. Remove aversive stimulus<br>7.6. Satiation<br>7.7. Exposure<br>7.8. Associative learning
10	**8. Repetition and substitution**<br>8.1. Behavioral practice/rehearsal<br>8.2. Behavior substitution<br>8.3. Habit formation<br>8.4. Habit reversal<br>8.5. Overcorrection<br>8.6. Generalisation of target behavior<br>8.7. Graded tasks
11	**9. Comparison of outcomes**<br>9.1. Credible source<br>9.2. Pros and cons<br>9.3. Comparative imagining of future outcomes
12	**10. Reward and threat**<br>10.1. Material incentive (behavior)<br>10.2. Material reward (behavior)<br>10.3. Non-specific reward<br>10.4. Social reward<br>10.5. Social incentive<br>10.6. Non-specific incentive<br>10.7. Self-incentive<br>10.8. Incentive (outcome)<br>10.9. Self-reward<br>10.10. Reward (outcome)<br>10.11. Future punishment
15	**11. Regulation**<br>11.1. Pharmacological support<br>11.2. Reduce negative emotions<br>11.3. Conserving mental resources<br>11.4. Paradoxical instructions
16	**12. Antecedents**<br>12.1. Restructuring the physical environment<br>12.2. Restructuring the social environment<br>12.3. Avoidance/reducing exposure to cues for the behavior<br>12.4. Distraction<br>12.5. Adding objects to the environment<br>12.6. Body changes
17	**13. Identity**<br>13.1. Identification of self as role model<br>13.2. Framing/reframing<br>13.3. Incompatible beliefs<br>13.4. Valued self-identify<br>13.5. Identity associated with changed behavior
18	**14. Scheduled consequences**<br>14.1. Behavior cost<br>14.2. Punishment<br>14.3. Remove reward<br>14.4. Reward approximation<br>14.5. Rewarding completion<br>14.6. Situation-specific reward<br>14.7. Reward incompatible behavior<br>14.8. Reward alternative behavior<br>14.9. Reduce reward frequency<br>14.10. Remove punishment
19	**15. Self-belief**<br>15.1. Verbal persuasion about capability<br>15.2. Mental rehearsal of successful performance<br>15.3. Focus on past success<br>15.4. Self-talk
19	**16. Covert learning**<br>16.1. Imaginary punishment<br>16.2. Imaginary reward<br>16.3. Vicarious consequences

Michie S, Richardson M, Johnston M, Abraham C, Francis J, Hardeman W, Eccles MP, Cane J, Wood CE. (2013). The Behavior Change Technique Taxonomy (v1) of 93 hierarchically clustered techniques: building an international consensus for the reporting of behavior change interventions, *Annals of Behavioral Medicine*, 2013;46(1): 81-95. doi: 10.1007/s12160-013-9486-6

Supplementary File S2: OVID Medline Search Strategy

1. (vaccin* and (hesitan* or refus* or confiden* or accept* or uptake* or adopt*)).mp. [mp=title, abstract, original title, name of substance word, subject heading word, floating sub-heading word, keyword heading word, organism supplementary concept word, protocol supplementary concept word, rare disease supplementary concept word, unique identifier, synonyms]
2. (ethnic group* or ethnic minorit* or minority group* or ethnic* or minorit* or race* or racial or Black* or African* or Asian* or South Asian* or Bangladeshi* or Pakistani* or Japanese or Chinese or Korean* or Arab* or BME or BAME or Roma* or Hispanic* or Caribbean* or people of color or person of color).mp. [mp=title, abstract, original title, name of substance word, subject heading word, floating sub-heading word, keyword heading word, organism supplementary concept word, protocol supplementary concept word, rare disease supplementary concept word, unique identifier, synonyms]
3. (Interven* or communicat* or train* or motiv* or strateg* or guid* or program* or support* or polic* or approach* or procedure* or plan* or engag*).mp. [mp=title, abstract, original title, name of substance word, subject heading word, floating sub-heading word, keyword heading word, organism supplementary concept word, protocol supplementary concept word, rare disease supplementary concept word, unique identifier, synonyms]
4. 1 and 2 and 3
5. (HPV or malaria or typhoid or cholera).mp. [mp=title, abstract, original title, name of substance word, subject heading word, floating sub-heading word, keyword heading word, organism supplementary concept word, protocol supplementary concept word, rare disease supplementary concept word, unique identifier, synonyms]
6. 4 not 5
7. limit 6 to (english language and humans)

Supplementary File S3: Quality Assessment (Risk of Bias) of included studies

Study type	First Author, Year	Clear aims	Appropriate study design	Sample justified	Population clearly defined	Sample represents target population	Appropriate selection process	Address non-responders	Risk factor and outcome measured	Risk factors and outcome measured correctly	Statistical significance measure	Methods sufficiently described	Basic data described	Response rate raise concerns	Non-responders described	Results internally consistent	All results presented	Discussions and conclusions justified by results	Limitations discussed	Funding or conflicts of interest declared	Ethical approval or consent	Summary	Grade
**Cross-Sectional**<br>(n=2, unique studies)<br>(n= 1, mixed methods - Skirrow et al. 2020)<br>(n = 1, merged study - Healy et al. 2009)	Greenfield et al. (2015)	Yes	Yes	Yes	Yes	Yes	Yes	Yes	Yes	No	Yes	Yes	Yes	No	NA	Yes	No	Yes	Yes	Yes	No	Yes = 15<br>No = 4<br>NA = 1	Moderate
	Healy et al. (2009)	Yes	Yes	Yes	Yes	Yes	Yes	Yes	Yes	Yes	Yes	Yes	Yes	No	No	Yes	Yes	Yes	No	No	Yes	Yes = 16<br>No = 4<br>Don't know= 0	Moderate
	Hoppe & Eckert (2011)	Yes	Yes	Yes	Yes	Yes	Yes	NA	Yes	Yes	Yes	No	Yes	No	NA	Yes	Don't know	Yes	Yes	No	Yes	Yes = 14<br>No = 3<br>Don't know= 1<br>NA = 2	Moderate
	Skirrow et al. (2020)	Yes	Yes	Yes	Yes	Yes	Yes	Yes	No	No	No	No	Yes	No	Yes	Yes	No	Yes	Yes	Yes	Yes	Yes = 14<br>No = 6<br>Can't tell= 0	Moderate

Study type	First Author, Year	Clear focus	Acceptable recruitment	Exposure accurately measured	Outcome accurately measured	Identified confounders	Confounders considered in design	Follow up completion	Follow up length sufficient	Study results	Result precision	Believe results	Results applicable to local population	Results fit other evidence	Study implications	Summary	Grade
**Cohort**<br>(n=4, unique studies)<br>(n = 3, merged study - Healy et al. 2011, 2012, 2015)	Broderick et al. (2018)	Yes	Yes	Can't tell	Can't tell	Can't tell	Yes	Yes	Yes	Yes	Yes	Yes	Can't tell	Yes	See Table 2 &	Yes = 9<br>No = 0<br>Can't tell = 4	Moderate
	Daniels et al. (2006)	Yes	Yes	Yes	Yes	Can't tell	Can't tell	Can't tell	Can't tell	See Table 2	See Table 2	Yes	Yes	Yes	See Table 2 & 5	Yes = 7<br>No = 0<br>Can't tell= 4	Moderate
	Healey et al. (2011)	Yes	Yes	Yes	Yes	No	No	Can't tell	Can't tell	See Table 2	See Table 2	Yes	Yes	Yes	See Table 2 & 5	Yes = 7<br>No = 2<br>Can't tell= 2	Moderate
	Healy et al. (2012)	Yes	Yes	Yes	Yes	No	No	Yes	Yes	See Table 2	See Table 2	Yes	Can't tell	Can't tell	See Table 2 & 5	Yes = 7<br>No = 2<br>Can't tell= 2	Moderate
	Healy et al. (2015)	Yes	Yes	Yes	Yes	Can't tell	Can't tell	Yes	Yes	See Table 2	See Table 2	Yes	Yes	Yes	See Table 2 & 5	Yes = 9<br>No = 0<br>Can't tell= 2	Moderate
	Hechter et al. (2019)	Yes	Yes	Yes	Yes	Yes	Yes	Yes	Yes	See Table 2	See Table 2	Yes	Yes	Yes	See Table 2 & 5	Yes = 11<br>No = 0<br>Can't tell= 0	High
	Howe et al. 2022	Yes	Yes	Yes	Yes	Yes	Yes	Yes	Yes	See Table 2	See Table 2	Yes	Yes	Yes	See Table 2 & 5	Yes = 11<br>No = 0<br>Can't tell= 0	High
	Traeger et al. (2006)	Yes	Yes	Yes	Yes	Can't tell	Can't tell	Can't tell	Can't tell	See Table 2	See Table 2	Yes	Yes	Yes	See Table 2 & 5	Yes = 7<br>No = 0<br>Can't tell= 4	Moderate

Study type	First Author, Year	Clearly research question	Participants randomised	All participants accounted	Participants blinded	Investigators blinded	Analyst blinded	Groups similar at start	Each group received the same care (besides intervention)	Effects of intervention reported	Estimated precision	Benefits of intervention outweigh the harms and costs	Results applicable to local population	Intervention provide greater than existing interventions	Summary	Grade
RCT<br>(n=5, unique studies)	Debroy et al (2023)	Yes	Yes	Yes	Can't tell	No	Can't tell	Yes	Yes	Yes	Yes	Yes	Yes	Yes	Yes = 10<br>No = 1<br>Can't tell = 2	Moderate
	Henrikson et al. (2015)	Yes	Yes	Yes	No	Can't tell	Yes	Yes	Yes	Yes	Yes	Yes	Yes	Yes	Yes = 11<br>No = 1<br>Can't tell= 1	High
	Humiston et al. (2011)	Yes	Yes	Yes	No	Yes	Can't tell	Yes	Yes	Yes	Yes	Yes	Yes	Yes	Yes = 11<br>No = 1<br>Can't tell= 1	High
	Nowalk et al. (2008)	Yes	Yes	Yes	Can't tell	Can't tell	Can't tell	Yes	Yes	Yes	Yes	Yes	Yes	Yes	Yes = 10<br>No = 0<br>Can't tell= 3	Moderate
	Sabnis et al (2003)	Yes	No	Yes	Can't tell	Can't tell	Can't tell	Yes	Yes	No	Yes	Yes	Yes	Yes	Yes = 8<br>No = 2<br>Can't tell= 3	Moderate

Study type	First Author, Year	Clear statement of aims	Methodology appropriate	Design appropriate	Recruitment strategy appropriate	Data collection addressed research issue	Relationship between researcher and participants considered	Ethical issues considered	Data analysis rigorous	Clear statement of findings	Value of research	Summary	Grade
**Qualitative**<br>**(n= 1, mixed methods)**	Skirrow et al. (2020)	Yes	Can't tell	Yes	Yes	Yes	No	Yes	No	Yes	See paper	Yes = 6<br>No = 2<br>Can't tell= 1	Moderate

Supplementary File S4: Identified intervention components mapped to relevant BCTs.

BCT Group	BCT	Brodeick et al. (2018)	Daniels et al. (2006)	Debroy et al. (2023)	Greenfield et al. (2015)	Healy et al. (2009, 2011, 2012, 2015)	Hechter et al. 2019	Henrikson et al. (2015)	Hoppe & Eckert (2011)	Howe et al. 2022	Humiston et al. (2011)	Nowalk et al. (2008)	Sabnis et al. (2003)	Skirrow et al. (2020)	Traeger et al. (2006)
**1. Goals and planning**	1.4. Action planning							Intervention clinics received training on a communication strategy , "Ask, Acknowledge, Advise,"							
**2. Feedback and monitoring**	2.1. Monitoring of behaviour by others without feedback		Prior to the patient's-visit with their physician, the nurse reviewed vaccination reminders, medical comorbidities and prior vaccination history to determine which patients should be offered pneumococcal vaccination.	A dashboard was presented relaying patient vaccination history and status					Use of stickers as prompts to monitor the vaccine acceptance or refusal of patients						
	2.2. Feedback on behaviour	Each provider was notified weekly by e-mail of which enrolled patients ... their immunization status, and personalized encouragement based on their performance.											Information on how many children could have been vaccinated that were not		
	2.4. Self-monitoring of outcome(s) of behaviour					All immunized women provided with an updated immunization record for future medical documentation.									
	2.5. Monitoring of outcome(s) of behaviour without feedback			Electronic record of vaccine registry updated daily used to easily identify the patients who had not yet received their vaccine					Electronic record of vaccine registry updated daily used to easily identify the patients who had not yet received their vaccine		Outcome of vaccinated or not using provider reminders, patient recall, and outreach to patients	Standing orders for nursing staff to screen adults and vaccinate; then mail fliers to age-eligible patients and telephoned high-risk patients			Vaccinations were documented electronically, and this automatically updated health summaries so that a patients vaccination was subsequently available throughout the service unit
	2.10 Monitoring of behaviour by others with feedback	Provider performance monitored and reported back via feedback emails.													

**3. Social support**	3.2. Social support (practical)			Modified electronic health record clinical reminder included talking points for providers to address vaccine hesitancy		- Nurses caring for postpartum women were available to answer questions<br>- Physician directors of the project also addressed any issues or questions from nurses and physicians.		- The "ask" step cues physicians to invite parental vaccine questions and concerns<br>- the acknowledge" step reinforces communication of respect and empathy for the parent's concerns and creation of a trusting environment<br>- Technical assistance provided	Pregnant women choose a Lead Maternity Carer (LMC) who supported them and answered queries on pregnancy, birth care, and infant care up to age six weeks.		Transportation assistance			Clinic promoted locally, and women could be referred by other midwives and doctors whilst attending routine antenatal appointments, self-refer, or be opportunistically approached by the vaccine midwives in the antenatal waiting rooms.	- Recommendations and mechanisms for acquiring vaccination<br>- public health nurses provided home visits and vaccinations, and vaccination information<br>- instructions were left for targeted individuals if they were not home.<br>- When the vaccine arrived, broadcast information on the tribal radio station about vaccine availability, groups at increased risk for influenza complications, and recommendations and mechanisms for acquiring vaccination
**4. Shaping knowledge**	4.1. Instruction on how to perform the behaviour	Providers received an "educational session … focused on the rationale, efficacy, and recommended uses of vaccination in patients with RA."		The intervention prompts primary care workers to recommend immunization and presents talking points to address patient uncertainty or refusal of the vaccine				The "advise" step prompts physicians to recommend immunization, educate about the benefits and risks of vaccines and vaccine-preventable disease, and end the consultation with a mutually agreed on action such as vaccinating or an appointment to discuss further.<br><br>A link to the study Web site that included a webinar version of the training, and technical assistance		Pharmacists could attend a training evening on maternal pertussis and influenza vaccinations.			Nurse training		'- When the vaccine arrived, broadcast information on the tribal radio station about vaccine availability, groups at increased risk for influenza complications, and recommendations and mechanisms for acquiring vaccination<br>- vaccination information and instructions were left for targeted individuals if they were not home.
	4.2. Information about Antecedents					- The rationale for cocooning was presented in obstetrical grand rounds and small group in-service-sessions.<br>- Healthcare providers educated about pertussis illness in infants and recommended									- Influenza and vaccine information provided to employees with message boards, verbal communications, and e-mail that promoted education and vaccination. plus website

						prevention strategy for young infants through obstetrical grand rounds and small group in-service sessions.<br>- Program education was incorporated into antenatal, baby-care, and breastfeeding classes.<br>- This education was supplemented, whenever possible, by a visit from our program nurse, who provided additional education to mothers and any visiting household contacts or caregivers.									
**5. Natural consequences**	5.1. Information about health consequences	In the education session, providers were given information on the rationale, efficacy and recommended use of vaccination for patients.			Culturally specific brochures targeted to parents of adolescents, professionally translated into Spanish, Tigrinya, Amharic, and Somali and featured photos of adolescents and families from these ethnic communities	Each postpartum woman received an information packet containing the Tdap vaccine information statement (VIS) and bilingual information leaflets (provided by the Texas Department of Health) on pertussis infection and the importance of Tdap vaccine for adults		- Intervention clinics received training on a novel communication strategy<br>- "advise" step prompts physicians to recommend immunization, educate about the benefits and risks of vaccines and vaccine-preventable disease	Presented data regarding the increased risk of H1N1 in pregnant women		Office visits were encouraged as a means to increase patient knowledge, address concerns, and clarify perceptions of health care	Immunization quiz for clinical and clerical staff	Providers were given pocket-sized laminate cards listing the recommended vaccine schedule		- Influenza and vaccine information provided to employees with message boards, verbal communications, and e-mail that promoted education and vaccination. plus website<br>- broadcast information on the tribal radio station about vaccine availability<br>- Public health nurses provided the same information to school administrators and to community members during home visits and community health classes (e.g., childbirth and parenting classes), a tribal elder conference, and health screening clinics
	5.2. Salience of consequences								Discussed the importance of preventative measures such as receiving the immunization, symptoms, and						

									receiving testing if our patients became symptomatic						
**7. Association**	7.1. Prompts/cues	An alert was entered into the EMR … displayed in red lettering on the main patient-specific screen.	A computerized reminder system of health maintenance information including pneumococcal vaccination status, was generated at each patient's visit.	Modified electronic health record clinical reminder that bundled together three adult vaccination reminders, presented patient vaccination history and included talking points for providers to address vaccine hesitancy.		Posters advocating Tdap vaccination were displayed prominently in antenatal, labour and delivery, and postpartum areas.	- Electronic reminders were posted to a patient's chart under a special tab. Physicians and nurses can click on the tab any time to review care gaps that need attention including immunization and screening tests due.<br><br>-An electronic alert is a ''hardstop'' clinical decision support tool, which pops up on the computer screen when a provider opens a patient's chart during a visit. Providers need to act on the alert (such as ordering a test or vaccination) or dismiss it manually. The reminders and alerts were triggered when a dose was due and removed from the patient's chart after the patient received the appropriate dose.	The "ask" step cues physicians to invite parental vaccine questions and concerns<br>The "advise" step prompts physicians to recommend immunization, educate about the benefits and risks of vaccines and vaccine-preventable disease, and end the consultation with a mutually agreed on action such as vaccinating or an appointment to discuss further.	- Used dated H1N1 vaccine acceptance or refusal stickers on the front of patient charts<br>- Created standing orders for the H1N1 vaccine administration<br>- Electronic vaccine registry was created by the nursing staff and updated daily,<br>- Created electronic schedule prompts to track patient's immunization status	Promotion to pharmacies and consumers for six months included Facebook posts, posters, and information for pharmacy staff and midwives	Provider reminders	- Standing orders for nursing staff to screen adults and vaccinate<br>- provider prompt for immunizations in its electronic medical record<br>- vaccination reminder on the front cover of the charts<br>- vaccination reminders in late summer as they left the office	Tagging	Vaccine midwives based in consulting rooms within the routine antenatal clinic. Some women were seen in the vaccine clinic on more than one occasion if they initially declined the vaccine on first contact.	Provided public information through radio broadcasts and brochures AND standing orders and prompts on patients' medical records permitted nurses to identify persons in targeted groups and vaccinate them immediately.
**9. Comparison of outcomes**	9.1. Credible source	Educational content "based on the most recent Advisory Committee on Immunization Practices and American College of Rheumatology guidelines.	If a patient agreed to be immunized, the nurse administered the vaccine; patients who declined were surveyed to assess their reasons for refusal and, according to the study protocol, would subsequently receive a firm recommendation from their physician during the visit.	The "ask" step cues primary care workers to address patient vaccine questions and concerns<br>- "Advise" step prompts primary care workers to recommend immunization, and address uncertainty and refusal of the vaccine	Community Health Promotors developed and conducted on-site presentations for HCPs at collaborating clinics and two additional high-volume clinics in the target communities.	Nursing personnel were particularly targeted because of their role as trusted advisors for new mothers and their potential to be powerful vaccine advocates		- The "ask" step cues physicians to invite parental vaccine questions and concerns<br>- "Advise" step prompts physicians to recommend immunization, educate about the benefits and risks of vaccines and vaccine-preventable disease		Universal maternity care and selected vaccinations available and pregnant women choose a Lead Maternity Carer (LMC) for the pregnancy, birth care, and infant care up to age six weeks. The LMC is usually an independent midwife or (less frequently) a public hospital		Posters encouraging vaccination were hung throughout the health centre, including patient posters in Vietnamese and Spanish.		Clinic was midwife-led who received training on maternal vaccine delivery and counselling. Vaccine midwives were senior, experienced midwives who had received specific training in the administration of vaccines and previously worked in both	Broadcast information on the tribal radio station about vaccine availability<br>Public health nurses provided the same information to school administrators and to community members during home visits and community health classes<br>(e.g., childbirth and parenting classes), a tribal

										midwife; and infrequently a general practitioner or obstetrician.				hospital obstetric and community midwifery roles.	elder conference, and health screening clinics.
	9.2. Pros and cons							The “advise” step prompts physicians to recommend immunization, educate about the benefits and risks of vaccines and vaccine-preventable disease, and end the consultation with a mutually agreed on action such as vaccinating or an appointment to discuss further.							
**10. Reward and threat**	10.1. Material incentive (behaviour)											Mailed reminders with a ‘‘free flu shot coupon’’ to all eligible adults			
	10.10. Reward (outcome)											- Sponsored contest for the most prolific vaccinator<br>- Vaccinators and vaccines received treat at the time of vaccination			
	10.2. Material reward (behaviour)						Outside providers to be reimbursed by the health plan for claims and capture of care delivered to members by electronic administrative data was reasonably assumed to be very comprehensive.					- Free flu shot			
	10.8. Incentive (outcome)						Vaccines were typically provided at no charge which is an incentive for members to receive immunizations within the system.					- Vaccination poster competition in which all who entered the health centre were eligible to vote<br>- Sponsored a contest for the			

												most prolific vaccinator			
**12. Antecedents**	12.1. Restructuring the physical environment	EMR alerts embedded into system navigation screens.						Each eligible physician, regardless of training attendance, received 6 months of monthly e-mail newsletters, a link to the study Web site that included a webinar version of the training, and technical assistance on request, such as responses to unusual parent questions about vaccine safety.	Placed an influenza prevention video in the waiting room that played continuously in 9 languages to increase the awareness of the influenza virus		Outreach to patients	- Immunization posters displayed in each examination room<br>- played immunization videos in the waiting room<br>- established walk-in influenza vaccine hours<br>- four influenza vaccination clinics in the surrounding neighbourhood<br>- scheduled nursing visits for patients to receive influenza vaccine		- Clinic was set up at NHS Trust to vaccinate pregnant women attending antenatal clinics.<br>- Clinic was midwife-led by two part-time dedicated ‘vaccine midwives’ who received training on maternal vaccine delivery and counselling.	Public health nurses provided influenza vaccinations in the community
	12.5. Adding objects to the environment		The opportunity for pneumococcal vaccination was offered before patients' appointments with their physicians.						Influenza prevention video played continuously in 9 languages<br><br>Patients were provided “flu packs” that contained masks, a digital thermometer, and hand antiseptic.	- Promotion to pharmacies and consumers included Facebook posts, posters, and information for pharmacy staff and midwives<br>- Vaccinations available in community pharmacies include influenza, Tdap and Maternal pertussis vaccines.	Adding vaccination to nurse visit - nurse already there for routine services		Providers were given pocket-sized laminate cards listing the recommended vaccine schedule, contraindications, and minimum interval schedule.		- Instructions were left for targeted individuals if they were not home<br>- Vaccinated in clinics, the emergency dept and the hospital<br>- Public health nurses provided influenza vaccinations in the community
**13. Identity**	13.2. Framing/reframing			Incorporated prompts to use presumptive language in the provider-intervention. Structured talking points were included to providers, to address specific forms of patient uncertainty or refusal	Culturally specific brochures featured photos of adolescents and families from these ethnic communities										
